# Audio Visual Assisted Therapy Aid for Refractory Auditory Hallucinations (AVATAR) therapy for voice hearers: systematic review and meta-analysis

**DOI:** 10.1192/bjo.2026.11014

**Published:** 2026-04-13

**Authors:** Felix Opper, Sebastian Henges, Pawel Weinstein, Dana Arnheim, Laura Fässler, Olivier Percie du Sert, Izabela Stefaniak, Michel Sabé, Louise Birkedal Glenthøj, Neil Thomas, Chih-Sung Liang, Brendon Stubbs, Kerem Böge

**Affiliations:** Department of Neuroscience and Psychiatry, Charité – Universitätsmedizin Berlin, corporate member of Freie Universität Berlin and Humboldt-Universität zu Berlin, Berlin, Germany; Department of Education and Psychology, Freie Universität Berlin, Berlin, Germany; Faculty of Psychology and Neuroscience, Maastricht University, Maastricht, Netherlands; Zachai Division of Psychiatry, Sheba Medical Center Israel and Sackler Faculty of Medicine, Tel Aviv University, Tel Aviv, Israel; Prevention and Early Intervention Program for Psychoses, Douglas Research Centre, Montreal, Quebec, Canada; Department of Psychiatry, McGill University, Montreal, Quebec, Canada; Faculty of Medicine, Lazarski University, Warsaw, Poland; Psychiatry Department, Faculty of Medicine, University of Geneva, Geneva, Switzerland; Division of Adult Psychiatry, Department of Psychiatry, University Hospitals of Geneva, Geneva, Switzerland; Department of Psychology, University of Copenhagen, Copenhagen, Denmark; VIRTU Research Group, Mental Health Center Copenhagen, Copenhagen University Hospital, Mental Health Services CPH, Copenhagen, Denmark; Centre for Mental Health and Brain Sciences, Swinburne University of Technology, Melbourne, Australia; Department of Psychiatry, Tri-Service General Hospital, National Defense Medical Center, Taipei, Taiwan; Department of Psychiatry, Beitou Branch, Tri-Service General Hospital, Taipei, Taiwan; Institute of Psychiatry, Psychology and Neuroscience (IoPPN), King’s College London, London, UK; Medical University Brandenburg-Theodor Fontane, Neuruppin, Germany; German Center of Mental Health (DZPG), Berlin/Potsdam, Germany

**Keywords:** AVATAR, digital intervention, voice hearing, schizophrenia spectrum disorders, virtual reality

## Abstract

**Background:**

Auditory verbal hallucinations (AVHs) are common and distressing symptoms across a range of psychiatric disorders, including schizophrenia spectrum disorders. Audio Visual Assisted Therapy Aid for Refractory Auditory Hallucinations (AVATAR) is an innovative therapeutic approach that facilitates dialogue with a digital avatar representing the voices that patients hear.

**Aims:**

This systematic review and meta-analysis aimed to assess the efficacy, tolerability and acceptability of AVATAR therapy in reducing voice-related symptoms.

**Method:**

Following preregistration, we conducted a systematic review and meta-analysis of controlled trials of AVATAR therapy in samples primarily diagnosed with schizophrenia spectrum disorders. PubMed, CINAHL, Embase, PsycInfo, ClinicalTrials.gov, ISRCTN and Web of Science were searched in March 2025. We assessed bias and certainty with the Cochrane Risk-of-Bias 2 tool and the GRADE approach. Random-effects models were used to synthesise outcomes.

**Results:**

Eight AVATAR trials (*N* = 978) were included. Compared with usual treatment, waitlist and active control groups, AVATAR therapy decreased the primary outcome of AVH severity at post-treatment (Hedges’ *g* = −0.40, 95% CI −0.54 to −0.25) and short-term follow-up (Hedges’ *g* = −0.25, 95% CI −0.40 to −0.10). AVH subscales showed small significant effect sizes at post-treatment (frequency: Hedges’ *g* = −0.38, 95% CI −0.52 to −0.24; distress: Hedges’ *g* = −0.32, 95% CI −0.46 to −0.18), which were maintained at short-term follow-up. The certainty of evidence was rated moderate for AVH severity at post-treatment. AVATAR therapy was largely tolerable and acceptable, with adverse events mostly unrelated to the treatment and a comparable drop-out rate to control groups.

**Conclusions:**

Findings suggest that AVATAR therapy is effective at reducing AVH symptoms. Considering heterogeneous control groups and less clear evidence for secondary outcomes and longer follow-ups, further research is warranted.

Auditory verbal hallucinations (AVHs), the experience of hearing voices without an external source,^1^ can occur across a wide range of psychiatric disorders, including schizophrenia spectrum disorders, affective disorders, borderline personality disorder, post-traumatic stress disorder, as well as in non-clinical populations.^2^ Despite their cross-diagnostic presence, AVHs are most commonly associated with schizophrenia spectrum disorders, where approximately 70% of patients experience them at some point during their lives.^3^ Phenomenologically, AVHs are highly heterogeneous, varying in content, emotional valence and perceived agency.^4^ They may be personified or non-personified, and experienced as persecutory, abusive, obscene, derogatory, threatening or critical, but also potentially helpful, affirming or inspirational.^3^

Although not inherently pathological, AVHs can become clinically relevant when they are experienced as intrusive, uncontrollable or malevolent.^5^ In such cases, AVHs are often associated with heightened distress, functional impairment and increased psychopathology.^6^

In the treatment of schizophrenia spectrum disorders, guidelines emphasise a multidisciplinary approach, with antipsychotic medication as a central component.^7–9^ Although antipsychotics are effective for a substantial proportion of patients,^10^ approximately 20–35% do not experience clinically meaningful improvement.^11–13^ Limitations are further underscored by high relapse rates upon medication discontinuation,^14,15^ and the risk of burdensome side-effects.^16^ Notably, around 30% of treatment-resistant symptoms involve persistent AVHs,^17–19^ highlighting the urgent need for additional, targeted interventions for individuals who continue to experience significant voice-related distress despite standard pharmacological care.

In addition, psychological interventions such as cognitive–behavioural therapy (CBT) are recommended.^7–9^ CBT has demonstrated small effects on psychotic symptoms in numerous domains, including overall positive^20^ and AVH-related symptoms.^21^ However, CBT shows relevant limitations including a large number of sessions required for effectiveness.^22^ More recently, a shift toward symptom-specific approaches has gained momentum, allowing for more personalised treatment strategies.^23^ For AVHs, relational therapies have emerged as a promising line of intervention, drawing on the person-like qualities of voices and conceptualising AVHs as embedded within a dynamic, relationship-like framework.^24^ Among these, the therapy intervention Audio Visual Assisted Therapy Aid for Refractory Auditory Hallucinations (AVATAR) represents a novel therapeutic development explicitly aimed at improving outcomes for individuals experiencing distressing and persistent voices.^25^

Initially developed by Julian Leff in 2008,^26^ AVATAR therapy enables real-time interactions with a digital representation of a person’s most dominant AVH. The approach involves the creation of a patient-designed digital avatar displayed either on a screen or through a virtual reality headset. A clinician then animates the avatar by voicing it according to the patient’s descriptions, and the avatar’s facial and head movements are synchronised to simulate natural speech. This simulated **‘**face-to-face’ interaction with the avatar functions as a form of exposure to anxiety-provoking stimuli,^27^ with the therapeutic aim of gradually increasing the individual’s sense of control, reducing fear-based appraisals and altering the relational dynamics with the voice. By enabling patients to assert themselves and challenge previously threatening voices, AVATAR therapy may reduce the distress associated with AVHs.^28^ In line with this, maladaptive appraisals of voices, such as beliefs about omnipotence or malevolence, have shown associations with voice-related distress, whereas more positive interpretations were modestly associated with reduced distress.^29^

Since its creation, AVATAR therapy has been evaluated in several clinical trials.^25^ Previous reviews have examined AVATAR therapy among virtual reality-based treatments in mental disorders or positive symptoms of schizophrenia spectrum disorders,^30,31^ but they have not provided a comprehensive perspective of AVATAR therapy for AVHs beyond its role within the broader context of virtual reality-based treatments. Two previous systematic reviews and meta-analyses have examined AVATAR trials and have reported promising effects on AVH-related symptoms.^32,33^ However, these were limited by the inclusion of few studies in the meta-analysis, by analysis of short follow-ups and by the lack of secondary aspects such as tolerability and acceptability of the treatment. These aspects are especially relevant for clinical decision-makers and guideline developers in evaluating the real-world utility of emerging therapies. Moreover, further high-quality clinical trials may have since been published, revealing the necessity for an updated review. In light of these limitations, we aimed to comprehensively assess the efficacy of AVATAR-based interventions for AVHs, including both clinical and functional outcomes, as well as their tolerability, acceptability and the overall quality of available evidence.

## Method

This systematic review and meta-analysis was preregistered at PROSPERO on 15 March 2025 (identifier CRD420251005545) and followed the Preferred Reporting Items for Systematic Reviews and Meta-Analyses (PRISMA) reporting guidelines (see Supplementary Tables 1 and 2 available at https://doi.org/10.1192/bjo.2026.11014).^34^

### Search strategy

On 21 March 2025, the publication databases Embase, PubMed and PsycINFO, as well as the grey literature and trial registration databases Web of Science Core Collection, CENTRAL, ClinicalTrials.gov and ISRCTN, were searched for relevant studies, to minimise the potential of publication bias. Indexing and general terms were applied in the search (see Supplementary Table 3).^35^ References of prior reviews and included articles were checked for additional studies, and experts were contacted for knowledge of further suitable trials. Duplicate removal and record screening were performed in the systematic review program Rayyan.^36^ F.O. and S.H. independently screened titles and abstracts for full-text screening eligibility with hierarchical criteria (see Supplementary Table 4),^37^ before independently completing the full-text screening in a spreadsheet. Corresponding authors of trial registrations and missing data were contacted weekly over the period of 4 weeks to request missing information. For disagreements during screenings, a third reviewer (K.B.) was consulted for discussion. Interrater reliability in the metric of Cohen’s *κ* was calculated using the DeltaMAN package for both screening stages.^38,39^

### Inclusion criteria

To be included, studies had to be published or unpublished, randomised controlled trials or controlled trials, investigating the efficacy of AVATAR or equivalent therapy in treating samples of participants aged ≥16 years who reported AVHs and were diagnosed with a schizophrenia spectrum disorder in >50% of cases, compared to any active or passive control group. Finally, studies had to report one of the following outcomes of interest.

The primary outcome of this study was the between-group difference of severity of voice symptoms at post-treatment measured with the Psychotic Symptom Rating Scales for Auditory Hallucinations (PSYRATS-AH^40^). Secondary outcomes included further voice-related (frequency and distress in the PSYRATS-AH), clinical (positive, negative, total psychotic, depressive and anxiety symptoms) and functional outcomes (social functioning and quality of life), as well as their follow-up assessments. We also indexed acceptability by examining within- and between-group drop-out rates, and tolerability from reports of treatment-related adverse events and symptom exacerbations. For multiple non-AVH measurements per study and outcome, the more primary measurement was included into the quantitative analysis (per study definition or earlier reference as an outcome). Follow-up measurements were categorised as short (12–23 weeks), medium (24–51 weeks) and long term (≥52 weeks) as a pragmatic approach reflecting common follow-up durations selected in meta-analyses of psychological intervention trials in schizophrenia spectrum disorders.^41,42^

We examined both treatment and study drop-out. Treatment drop-out was identified by any participant who did not complete the treatment post-randomisation.^43^ The criteria for having finished a treatment followed that of each study. In contrast, study drop-out was characterised by participants lost to post-treatment assessments regardless of reason.

### Data extraction, synthesis and effect sizes

Data was independently extracted by F.O. and S.H., and cross-checked by D.A. The data extraction started on 2 April 2025. Meta-analysis was performed when at least two effect sizes could be pooled per analysis. Although AVATAR therapy approaches are similar, we anticipated differences in control groups, leading us to the use of random-effects models, which allow for heterogeneous treatment effects.^44^

The metric of Hedges’ *g* was used to report the standardised mean difference for continuous outcomes and aggregate outcomes of overlapping constructs using different scales.^45^ Negative effect sizes described outcomes with a lower mean score in the AVATAR groups relative to the control groups, with positive values indicating the opposite. Values of 0.2, 0.5 and 0.8 were considered the thresholds for low, medium and large effect sizes, respectively.^46^ Proportion and risk ratio were calculated for within- and between-group drop-out effect sizes. Drop-out proportions were calculated with generalised linear-mixed models.^47^ Risk ratios >1 portrayed an increased risk in AVATAR relative to control groups. To adjust for zero-case studies, 0.5 cases of drop-out were added to each study’s risk table containing at least one instance of zero drop-outs in either group.^48^ For three-arm studies, the sample sizes of the control groups were halved to include both AVATAR groups, per the suggestions in the Cochrane Handbook.^49^

The presence of heterogeneity was tested with Cochran’s *Q*-test and interpreted as Higgins’ *I* ^*2*^ *-statistic.* ^50,51^ Percentages of 25%, 50% and 75% were considered low, moderate and high levels of heterogeneity, respectively. All analyses were performed in R version 2025.05.1+513 in RStudio for macOS, using the metafor and Tidyverse packages, and were visualised with forest plots.^52–54^ To exploratively assess the robustness of effects on AVH symptoms, subgroup analyses of studies with low and high risks of bias were performed. Additionally, for AVH symptoms, analyses by control group (treatment as usual versus treatment as usual plus waitlist versus active control group) were performed with subgroup analyses and mixed-effects meta-regression. Results with significant heterogeneity were examined with jackknife analyses.^55^ An *α*-level of 0.05 was considered for all analyses.

### Bias assessments, publication bias and quality of evidence

F.O. and S.H. independently assessed risk of bias with the Revised Cochrane Risk-of-Bias Tool for Randomized Trials (RoB-2^56^), and differences were discussed with a third reviewer, K.B. Bias was rated under consideration of the primary outcome of AVH severity. The risk of bias was visualised using the robvis app.^57^ Publication bias may occur when articles remain unpublished because of unwanted study results.^58^ For main analyses, funnel plots were plotted for its assessment and asymmetry was tested with Egger’s tests.^59^ Asymmetric analyses were eligible to be corrected with the trim-and-fill procedure.^60^ F.O. characterised the certainty of meta-analytic evidence according to the Grading of Recommendations, Assessment, Development and Evaluation (GRADE) criteria, using GRADEpro online software.^61,62^

## Results

### Study selection

The literature search identified 556 records (see the PRISMA flow diagram in Supplementary Fig. 1). After duplicate removal and dual screenings, a total of seven peer-reviewed articles and one letter to the editor were included. We contacted authors of trial registrations and conference abstracts, although no ongoing trials were able to provide includable information concerning relevant outcomes for this study. Abstract and full-text screening exclusion reasons can be viewed in Supplementary Tables 4 and 5. The original AVATAR trial included a small number of participants under 16 years of age,^25^ formally conflicting with our inclusion criteria. Given this small proportion and comparable mean age and standard deviation to other included samples, the study was retained. The title and abstract as well as full-text screening interrater reliabilities revealed Cohen’s *κ* = 0.95 and 0.93, respectively, which can be considered satisfactory.^63^

### Study and sample characteristics

The eight randomised and controlled studies included nine relevant comparisons (Garety et al^64^ included two AVATAR groups) and *n* = 554 participants in the AVATAR therapy groups and *n* = 424 in the control groups. Of note is that three studies were partial crossover trials,^25,65,66^ with only the period before crossover included in the analyses. The post-treatment measurement varied from 7 to 16 weeks (mean 9.88 weeks). Each study that performed a follow-up assessment did so at 12–13-week follow-up, and one study additionally assessed at 24- and 52-week follow-ups.^67^ All participants received treatment as usual, typically including antipsychotic medication. Control groups consisted of treatment as usual alone,^64^ treatment as usual plus waitlist,^25,65,66^ or treatment as usual with an additional active control condition (CBT,^67,68^ supportive counselling,^26^ enhanced treatment as usual with supportive counselling sessions^69^). The studies with an additional active control condition were categorised as active control groups in the subgroup analysis. The range of treatment durations was 6 to 9 weeks or sessions except for AVATAR-EXT,^64^ an extension of AVATAR in 12 weekly sessions. Samples consisted of persons with schizophrenia spectrum disorders reporting treatment-resistant, chronic or persisting AVHs, although three studies included small subsamples of other mental disorders.^26,64,66^ Demographic and clinical outcomes, as well as specific study characteristics, can be found in Table 1. Additional information (protocols, criteria, settings, diagnosis distributions, participant ages and funding) is provided in Supplementary Table 6, whereas Supplementary Table 7 describes additional information received from study authors.

**Table 1:** Study characteristics

Study and country	Experimental and control group conditions	*N*_Experimental group, control group_	Duration	Sample type and diagnoses	Outcome measures of interest	Assessments (after T0)	Study design
Craig et al, 2018 (UK)^26^	AVATAR + TAU	75 *v*. 75	Six weekly sessions at 50 min, extendable up to ten sessions	Treatment-resistant AVHs; schizophrenia, schizoaffective disorder, bipolar disorder, unspecified psychosis, depression with psychotic symptoms	Voice severity, frequency, distress, positive symptoms, negative symptoms, depression, quality of life	T0 T1 = 12 weeks T2 = 24 weeks	RCT
	Supportive counselling + TAU						
Dellazizzo et al, 2021 (Canada)^67^	AVATAR (virtual reality) + TAU	37 *v*. 37	Nine weekly sessions at 60 min	Treatment-resistant AVHs; schizophrenia, schizoaffective disorder	Voice severity, frequency, distress, total psychotic symptoms, positive symptoms, negative symptoms, depression, quality of life	T0 T1 = 9 weeks T2 = 22 weeks T3 = 35 weeks T4 = 61 weeks	RCT
	CBT + TAU						
Garety et al, 2024 (UK)^64^	AVATAR extended + TAU	114	12 sessions at 45–60 min	AVHs persisting for more than 6 months; schizophrenia, persistent delusional disorders, acute and transient psychotic disorders, induced delusional disorder, schizoaffective disorders, other non-organic psychotic disorders, unspecified non-organic psychosis, bipolar affective disorder, severe depressive episode with psychotic symptoms	Voice distress, frequency, total severity, anxiety, depression	T0 T1 = 16 weeks T2 = 28 weeks	RCT
	AVATAR brief + TAU	116	Six sessions at 45–60 min				
	TAU	115					
Leff et al, 2013 (UK)^25^	AVATAR + TAU	14 *v*. 12	7 weeks, six sessions at 30 min	Treatment-resistant AVHs; schizophrenia	Voice severity, depression, voice frequency, distress	T0 T1 = 7 weeks	RPCT
	TAU + waitlist						
Liang et al, 2022 (China)^68^	AVATAR (virtual reality + motion capture) + TAU	32 *v*. 33	Seven to nine weekly sessions at 60 min	Treatment-resistant AVHs; schizophrenia	Voice severity, distress, frequency, total psychotic symptoms, positive symptoms, negative symptoms, quality of life, depression, anxiety	T0 T1 = 7–9 weeks T2 = 19–21 weeks	RCT
	CBT + TAU						
Percie du Sert et al, 2018 (Canada)^65^	AVATAR (virtual reality) + TAU	12 *v*. 7	Seven weekly sessions at 45 min, extendable up to 11 sessions	Treatment-resistant AVHs; schizophrenia, schizoaffective disorder	Voice severity, frequency, distress, total psychotic symptoms, positive symptoms, negative symptoms, depression, quality of life	T0 T1 = 7 weeks	RPCT
	TAU + waitlist						
Smith et al, 2025 (Denmark)^69^	AVATAR (virtual reality) + TAU	140 *v*. 131	12 weeks, seven weekly to bi-weekly sessions at 60 min; two booster sessions after post-treatment	Treatment-resistant AVHs; schizophrenia, persistent delusional disorders, schizoaffective disorder, other non-organic psychotic disorders	Voice severity, frequency, distress, social functioning, positive symptoms, negative symptoms, depression	T0 T1 = 12 weeks T2 = 24 weeks	RCT
	Enhanced TAU (supportive counselling)						
Stefaniak et al, 2019 (Poland)^66^	AVATAR + TAU	14 *v*. 14	Eight sessions at 50 min	Chronic AVHs; schizophrenia; paranoid personality disorder	Positive symptoms, voice severity, frequency, distress	T0 T1 = not reported (likely 8 weeks)	RPCT
	TAU + waitlist						

*N*, sample size; T0, baseline; AVATAR, Audio Visual Assisted Therapy Aid for Refractory Auditory Hallucinations; TAU, treatment as usual; AVH, auditory verbal hallucinations; T1, post-treatment; T2, follow-up; RCT, randomised controlled trial; RPCT, randomised partial crossover trial.

Sample sizes given at baseline.

### AVATAR therapy protocols

The treatment protocols showed differences. Treatment began with the creation of a digital avatar, except in Stefaniak et al,^66^ which used a standardised avatar. Although some protocols enabled viewing the avatar via a virtual reality headset,^65,67–69^ others presented the avatar on a computer screen,^25,26,64,66^ and one used full-face and body motion capture of the therapist to increase the feeling of presence and immersion.^68^ The participants usually interacted with an avatar voiced by the therapist in real time, although in Stefaniak et al,^66^ a pre-programmed avatar was used, which the therapist and participant both interacted with.

Post-creation sessions included a therapeutic session preparation phase, followed by exposure to distressing utterances of the avatar, during which the participant was encouraged respond assertively.^26^ As the treatment progressed, the avatar’s verbalisations gradually became less abusive and more supportive to reflect the participants’ increase in control. Later sessions were designated for future outlook and relapse prevention. As an extension of this framework, AVATAR-EXT provided six additional sessions, where participants discussed the trauma, marginalisation and social exclusion background of their AVHs with the therapist before avatar exposure.^64^ Half of the studies provided recordings of the sessions, which could be listened to as homework to increase transferral to daily life.^25,26,64,68^

### Risk of bias

**Fig. 1:**
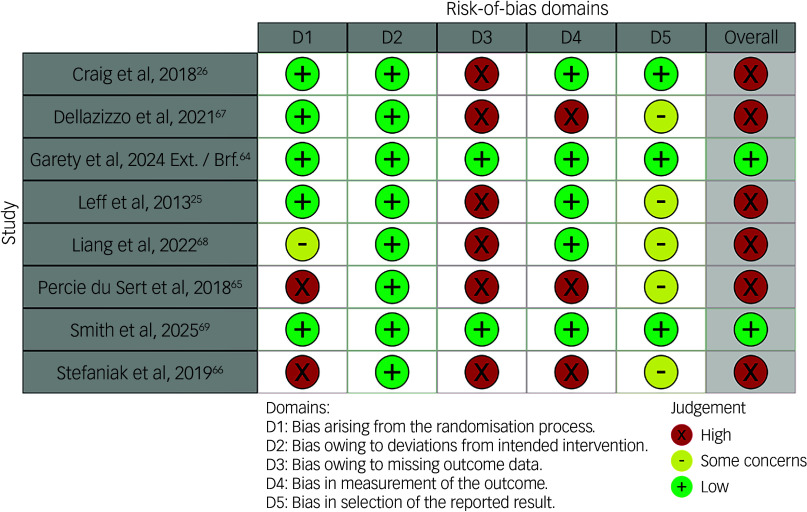
Risk-of-bias assessments. Ext., extended; Brf., brief.

The risk-of-bias assessments are displayed in Fig. 1, and the results of the bias assessment questions can be found in Supplementary Table 8. Overall, two studies were rated with low risk of bias,^64,69^ and the remaining six studies high overall risk of bias.^25,26,65–68^ Importantly, the two studies rated as having low risk of bias accounted for the majority (63%) of participants.

### Meta-analysis and systematic review results

Although control groups varied across studies, a main analysis of all studies was performed. Treatments for schizophrenia spectrum disorders differ depending on the setting, availability and severity of symptoms.^70–72^ Consequently, this approach provided greater external validity, but may have induced heterogeneity. Subgroup analyses by specific control group type were conducted to complement this approach. Forest plots of all secondary outcomes can be found in Supplementary Figs 2–6, the assessments of the certainty of evidence according to GRADE criteria can be found in Supplementary Table 9 and information on which scales were included can be found in Supplementary Table 10.

### Primary and secondary AVH outcomes: AVH severity, frequency and distress

**Fig. 2:**
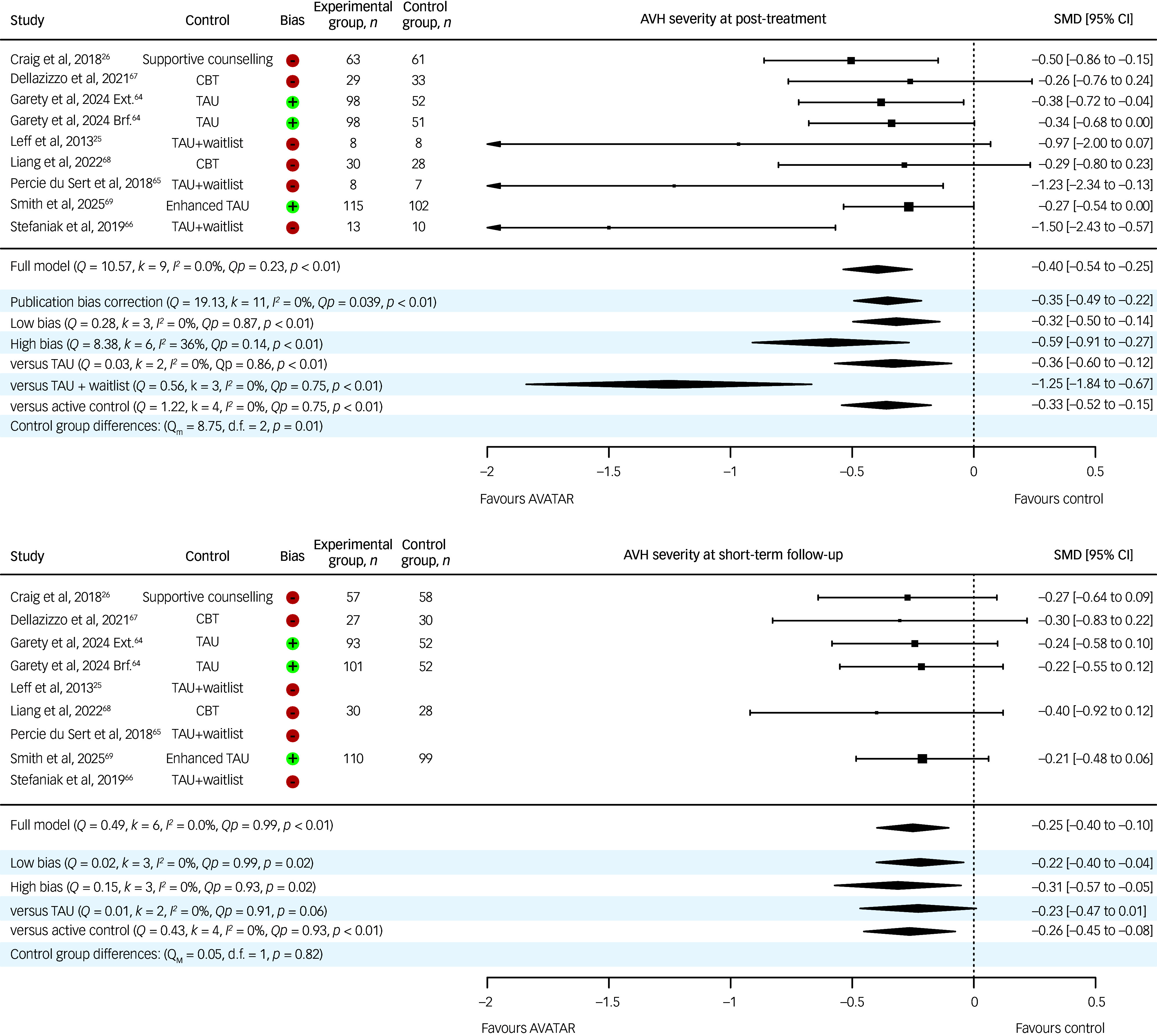
Forest plot showing meta-analytic results for AVH severity at post-treatment and follow-up. Negative SMDs portray smaller means in AVATAR compared with control groups. ⊕, Low bias; ⊖, high bias; AVATAR, Audio Visual Assisted Therapy Aid for Refractory Auditory Hallucinations; AVH, auditory verbal hallucination; Brf., brief; CBT, cognitive–behavioural therapy; Ext., extended; *I* ^2^, Higgins’ heterogeneity statistic; *k*, number of comparisons; *n,* sample size; *Q*, Cochran’s *Q*-statistic; *Qp*, Cochran’s *Q p*-value; SMD, standardised mean difference in Hedges’ *g*; TAU, treatment as usual.^66^

Table 2 presents the meta-analytic results for voice-related outcomes alongside the corresponding certainty of evidence, and Table 3 shows subgroup and moderation analyses by subgroup and bias. Each study assessed AVH severity, frequency and distress at pre- and post-treatment. Short-term 12- to 13-week follow-up assessments were performed in all but three studies.^25,65,66^ The pooled effect size on AVH severity at post-treatment was small, homogeneous and favoured AVATAR (Hedges’ *g* = −0.40, 95% CI −0.54 to −0.25, *p* < 0.001). This effect was moderated by control group (*p* = 0.01), although each subgroup favoured AVATAR. At short-term follow-up, the effect size remained small (Hedges’ *g* = −0.25, 95% CI −0.40 to −0.10, *p* < 0.001) and homogeneous. This effect was not moderated by control group (*p* = 0.82), but only the active control subgroup significantly favoured AVATAR. Medium- and long-term effect sizes were derived from only one study, and were small and non-significant. Studies rated with a low risk of bias revealed similar results at both post-treatment and short-term follow-up. Forest plots for AVH severity are displayed in Fig. 2. The certainty of evidence was rated moderate for post-treatment and low for the short-term-follow-up, whereas medium- and long-term follow-ups were rated very low.

**Table 2:** Meta-analytic results for auditory verbal hallucination-related outcomes

Outcome	Timeframe/analysis	Comparisons	*N*_Experimental group, control group_	*I* ^2^ *%*	*I* ^2^ *p*-value	Hedges’ *g* [95% CI]	Effect *p*-value	GRADE rating^a^
AVH severity	T1 (post-treatment)	9	462 *v*. 352	0	0.23	−0.40 [−0.54, −0.25]	<0.001***	Moderate
	T1 Publication bias correction	11	Not applicable	0	0.04*	−0.35 [−0.49, −0.22]	<0.001***	Not applicable
	T2 (short-term follow-up)	6	418 *v*. 319	0	0.99	−0.25 [−0.40, −0.10]	<0.001***	Low
	T3 (medium-term follow-up)	1	13 *v*. 20	Not applicable	Not applicable	−0.30 [−1.00, 0.40]	0.40	Very low
	T4 (long-term follow-up)	1	13 *v*. 17	Not applicable	Not applicable	−0.26 [−0.99, 0.46]	0.48	Very low
AVH frequency	T1 (post-treatment)	9	462 *v*. 352	0	0.13	−0.38 [−0.52, −0.24]	<0.001***	High
	T2 (short-term follow-up)	6	421 *v*. 320	0	0.61	−0.34 [−0.49, −0.19]	<0.001***	Low
	T3 (medium-term follow-up)	1	13 *v*. 20	Not applicable	Not applicable	−0.33 [−1.03, 0.37]	0.36	Very low
	T4 (long-term follow-up)	1	13 *v*. 17	Not applicable	Not applicable	−0.40 [−1.12, 0.33]	0.29	Very low
AVH distress	T1 (post-treatment)	9	462 *v*. 352	0	0.09	−0.32 [−0.46, −0.18]	<0.001***	Low
	T1 Publication bias correction	11	Not applicable	0	0.005**	−0.28 [−0.41, −0.14]	<0.001***	Not applicable
	T2 (short-term follow-up)	6	419 *v*. 320	0	0.94	−0.20 [−0.35, −0.06]	0.007**	Very low
	T3 (medium-term follow-up)	1	13 *v*. 20	Not applicable	Not applicable	−0.21 [−0.91, 0.49]	0.55	Very low
	T4 (long-term follow-up)	1	13 *v*. 17	Not applicable	Not applicable	−0.18 [−0.90, 0.54]	0.62	Very low

*N*, sample size; *I* ^2^, heterogeneity statistic; GRADE, Grading of Recommendations, Assessment, Development and Evaluation; AVH, auditory verbal hallucinations; T1, post-treatment; T2, follow-up at 12 weeks; T3, follow-up at 24 weeks; T4, follow-up at 52 weeks.

Negative effect sizes described lower scores in intervention groups.

a. GRADE ratings^73^: High: a lot of confidence that the true effect lies close to that of the estimated effect. Moderate: moderate confidence in the estimated effect. The true effect is likely to be close to the estimated effect, but there is a possibility that it is substantially different. Low: limited confidence in the estimated effect. The true effect might be substantially different from the estimated effect. Very low: very little confidence in the estimated effect. The true effect is likely to be substantially different from the estimated effect.

*p < 0.05; **p < 0.01; ***p < 0.001.

**Table 3:** Meta-analytic subgroup and moderator results for auditory verbal hallucination-related outcomes

Outcome	Timeframe/analysis	Comparisons	*N*_Experimental group, control group_	*I* ^*2*^ *%*	*I* ^*2*^ *p*-value	Hedges’ *g* [95% CI]	Effect *p*-value
AVH severity	T1 Low bias	3	311 *v*. 205	0	0.87	−0.32 [−0.50, −0.14]	<0.001***
	T1 High bias	6	151 *v*. 147	36	0.14	−0.59 [−0.91, −0.27]	<0.001***
	T1 TAU control	2	196 *v*. 103	0	0.86	−0.36 [−0.60, −0.12]	0.003**
	T1 TAU + waitlist control	3	29 *v*. 25	0	0.75	−1.25 [−1.84, −0.67]	<0.001***
	T1 Active control	4	237 *v*. 224	0	0.75	−0.33 [−0.52, −0.15]	<0.001***
	T1 Control group comparison	9	462 *v*. 352	0	0.94	Not applicable	0.01*
	T2 Low bias	3	304 *v*. 203	0	0.99	−0.22 [−0.40, −0.04]	0.02*
	T2 High bias	3	114 *v*. 116	0	0.93	−0.31 [−0.57, −0.05]	0.02*
	T2 TAU control	2	194 *v*. 104	0	0.91	−0.23 [−0.47, 0.01]	0.06
	T2 Active control	4	224 *v*. 215	0	0.93	−0.26 [−0.45, −0.08]	0.006**
	T2 Control group comparison	6	418 *v*. 319	0	0.98	Not applicable	0.82
AVH frequency	T1 Low bias	3	311 *v*. 205	0	0.74	−0.34 [−0.52, −0.16]	<0.001***
	T1 High bias	6	151 *v*. 147	58	0.04*	−0.46 [−0.86, –0.06]	0.02*
	T1 TAU control	2	196 *v*. 103	0	0.59	−0.39 [−0.63, −0.15]	0.002**
	T1 TAU + waitlist control	3	29 *v*. 25	62	0.07	−0.79 [−1.73, 0.15]	0.10
	T1 Active control	4	237 *v*. 224	30	0.21	−0.32 [−0.55, −0.09]	0.007**
	T1 Control group comparison	9	462 *v*. 352	13	0.12	Not applicable	0.32
	T2 Low bias	3	307 *v*. 204	0	0.54	−0.34 [−0.52, −0.16]	<0.001***
	T2 High bias	3	114 *v*. 116	0	0.31	−0.33 [−0.59, −0.07]	0.01*
	T2 TAU control	2	194 *v*. 104	0	0.46	−0.41 [−0.65, −0.17]	<0.001***
	T2 Active control	4	227 *v*. 216	0	0.48	−0.29 [−0.48, −0.11]	0.002**
	T2 Control group comparison	6	421 *v*. 320	0	0.55	Not applicable	0.45
AVH distress	T1 Low bias	3	311 *v*. 205	0	0.85	−0.26 [−0.44, −0.08]	0.004**
	T1 High bias	6	151 *v*. 147	65	0.03*	−0.55 [−0.99, –0.10]	0.02*
	T1 TAU control	2	196 *v*. 103	0	0.86	−0.31 [−0.55, −0.07]	0.01*
	T1 TAU + waitlist control	3	29 *v*. 25	33	0.23	−1.13 [−1.85, –0.42]	0.002**
	T1 Active control	4	237 *v*. 224	0	0.42	−0.25 [−0.44, −0.07]	0.007**
	T1 Control group comparison	9	462 *v*. 352	0	0.44	Not applicable	0.02*
	T2 Low bias	3	305 *v*. 204	0	0.94	−0.17 [−0.35, 0.01]	0.06
	T2 High bias	3	114 *v*. 116	0	0.67	−0.27 [−0.53, –0.01]	0.04*
	T2 TAU control	2	194 *v*. 104	0	0.99	−0.14 [−0.38, 0.10]	0.24
	T2 Active control	4	225 *v*. 216	0	0.83	−0.24 [−0.43, −0.05]	0.01*
	T2 Control group comparison	6	419 *v*. 320	0	0.93	Not applicable	0.53

*N ,* sample size; *I* ^2^, heterogeneity statistic; AVH, auditory verbal hallucinations; T1, post-treatment; TAU, treatment as usual; T2, follow-up at 12–23 weeks; T3, follow-up at 24–51 weeks; T4, follow-up at 52 weeks.

Negative effect sizes described lower scores in intervention groups.

*p < 0.05; **p < 0.01; ***p < 0.001.

For AVH frequency, the pooled effect size at post-treatment was small, favoured AVATAR (Hedges’ *g* = −0.38, 95% CI −0.52 to −0.24, *p* < 0.001) and was homogeneous. Of the control group subgroup analyses, all but the AVATAR versus the treatment as usual plus waitlist group favoured AVATAR. The effect size at short-term follow-up remained small (Hedges’ *g* = −0.34, 95% CI −0.49 to −0.19, *p* < 0.001), significantly favoured AVATAR across subgroups and was homogeneous. Medium- and long-term follow-up effect sizes were derived from one study and were small and non-significant. Studies rated with a low risk of bias revealed similar results at both time points. The certainty of evidence was rated high for post-treatment and low for short term follow-up, whereas medium- and long-term follow-ups were rated very low.

For AVH distress, the pooled effect size at post-treatment was medium, significant (Hedges’ *g* = −0.32, 95% CI −0.46 to –0.18, *p* < 0.001) and homogeneous. This effect was moderated by control group (*p* = 0.02), but each of the subgroups significantly favoured AVATAR. The pooled small effect size at short-term follow-up favoured AVATAR (Hedges’ *g* = −0.20, 95% CI −0.35 to −0.06, *p* = 0.007) and was homogeneous. Only the active control subgroup favoured AVATAR at short-term follow-up. Medium- and long-term follow-up effect sizes were derived from one study and were negligible to small and non-significant. Studies rated with a low risk of bias revealed similar results at post-treatment, but were non-significant at short-term follow-up. The certainty of evidence was rated low for post-treatment and very low for short-term follow-up, whereas medium- and long-term follow-ups were rated very low.

### Secondary outcomes: clinical and functional outcomes

Table 4 presents the results of meta-analyses performed for secondary clinical outcomes. PSYRATS subscales measured by one study^64^ were aggregated according to the recommendations of Borenstein,^74^ assuming a correlation of 0.34 between delusions and hallucinations.^75^ For positive symptoms, small and negligible effect sizes significantly favoured AVATAR at post-treatment and short-term follow-up, respectively. In contrast, effect sizes for total psychotic and negative symptoms did not significantly favour either group at either time point. Small effect sizes significantly favoured AVATAR for anxiety and depressive symptoms at post-treatment, but remained significant only for anxiety symptoms at short-term follow-up. No significant between-group effects were observed for quality of life at either time point. Social functioning was measured by only one study,^69^ and was therefore not meta-analytically aggregated. The removal of a singular outlier^67^ at post-treatment, specifically for quality of life, reduced heterogeneity without altering the conclusion of the statistical tests. Medium- and long-term follow-up assessments were derived from one study and were non-significant for each outcome.

**Table 4:** Meta-analytic results for clinical and functional outcomes

Outcome	Timeframe/analysis	Comparisons	*N*_Experimental group, control group_	*I* ^*2*^ %	*I* ^*2*^ *p*-value	Hedges’ *g* [95% CI]	Effect *p*-value
Total psychotic symptoms	T1 (post-treatment)	3	67 *v*. 68	0	0.61	−0.20 [−0.54, 0.14]	0.26
	T2 (short-term follow-up)	2	57 *v*. 58	0	0.70	−0.17 [−0.54, 0.19]	0.35
	T3 (medium-term follow-up)	1	13 *v*. 20	Not applicable	Not applicable	0.26 [−0.45, 0.96]	0.47
	T4 (long-term follow-up)	1	13 *v*. 17	Not applicable	Not applicable	0.00 [−0.72, 0.72]	1
Positive symptoms	T1 (post-treatment)	8	442 *v*. 341	0	0.47	−0.29 [−0.43, −0.16]	<0.001***
	T2 (short-term follow-up)	6	407.5 *v*. 315.5^a^	0	0.64	−0.16 [−0.30, −0.03]	0.02*
	T3 (medium-term follow-up)	1	13 *v*. 20	Not applicable	Not applicable	0.29 [−0.41, 0.99]	0.42
	T4 (long-term follow-up)	1	13 *v*. 17	Not applicable	Not applicable	0.22 [−0.50, 0.95]	0.55
Negative symptoms	T1 (post-treatment)	5	245 *v*. 231	0	0.96	0.09 [−0.09, 0.27]	0.31
	T2 (short-term follow-up)	4	227 *v*. 216	0	0.90	0.00 [−0.19, 0.19]	0.99
	T3 (medium-term follow-up)	1	13 *v*. 20	Not applicable	Not applicable	0.14 [−0.56, 0.84]	0.69
	T4 (long-term follow-up)	1	13 v. 17	Not applicable	Not applicable	0.07 [−0.65, 0.79]	0.85
Depressive symptoms	T1 (post-treatment)	8	433 *v*. 332	46	0.06	−0.25 [−0.47, −0.04]	0.02*
	T2 (short-term follow-up)	6	400 *v*. 310	21	0.30	−0.13 [−0.30, 0.05]	0.15
	T3 (medium-term follow-up)	1	13 *v*. 20	Not applicable	Not applicable	0.70 [−0.02, 1.42]	0.06
	T4 (long-term follow-up)	1	13 *v*. 17	Not applicable	Not applicable	0.31 [−0.42, 1.03]	0.41
Anxiety symptoms	T1 (post-treatment)	4	272 *v*. 182	0	0.60	−0.45 [−0.65, −0.26]	<0.001***
	T2 (short-term follow-up)	4	260 *v*. 180	0	0.56	−0.24 [−0.43, −0.05]	0.01*
Quality of life	T1 (post-treatment)	4	128 *v*. 127	82	0.007**	0.33 [−0.33, 0.99]	0.33
	T1 Removal of outlier^67^	3	99 *v*. 94	58	0.09	0.52 [−0.00, 1.05]	0.05
	T2 (short-term follow-up)	3	114 *v*. 113	0	0.41	0.15 [−0.11, 0.42]	0.25
	T3 (medium-term follow-up)	1	13 v. 20	Not applicable	Not applicable	−0.31 [−1.01, 0.39]	0.39
	T4 (long-term follow-up)	1	13 *v*. 17	Not applicable	Not applicable	−0.40 [−1.13, 0.33]	0.28
Social functioning	T1 (post-treatment)	1	114 *v*. 102	Not applicable	Not applicable	−0.04 [−0.31, 0.22]	0.74
	T2 (short-term follow-up)	1	112 *v*. 99	Not applicable	Not applicable	0.04 [−0.23, 0.31]	0.77

*I* ^*2*^ , heterogeneity statistic; T1, post-treatment; T2, follow-up at 12 weeks; T3, follow-up at 24 weeks; T4, follow-up at 52 weeks; AVATAR, Audio Visual Assisted Therapy Aid for Refractory Auditory Hallucinations.

Negative effect sizes described lower means in AVATAR groups.

a. Decimals are because of aggregation of subscales.

*p < 0.05; **p < 0.01; ***p < 0.001.

### Secondary outcomes: tolerability and acceptability

Each study reported tolerability aspects concerning treatment-related adverse events or exacerbations, except for Leff et al.^25^ Adverse events and exacerbations were non-standardised, making narrative review necessary. In Percie du Sert et al,^65^ 1 participant out of 12 received additional counselling during early treatment, because of transient symptom exacerbations. Additionally, each of the four participants who dropped out did so because of anxiety symptom exacerbations in early virtual reality sessions. Three studies stated that any adverse events were not attributable to AVATAR or the control group.^26,67,68^ In Garety et al,^64^ 1 case of hospital admission in AVATAR (out of 116) and 4 cases in AVATAR-EXT (out of 114) could not be ruled out from possibly being attributable to the treatments. In Smith et al,^69^ of 140 participants, five cases of hospital admission and one case of self-harm were considered potentially related to the AVATAR treatment because of worsening of AVHs. AVH symptom increases occurred in 52 participants during early exposure, which gradually declined over the course of the therapy. Furthermore, 40 participants reported needing additional time to manage anxiety during virtual reality immersion. Finally, Stefaniak et al^66^ reported one case of hospital admission among 14 in the AVATAR group, although causal connections to the treatment were not reported.

Each study reported on treatment drop-outs in AVATAR and five reported rates in control groups. As can be seen in Table 5, the overall aggregated proportion of treatment drop-out in AVATAR was 24% with moderate heterogeneity. In control groups, the aggregated proportion of treatment drop-out was 18%, with moderate heterogeneity. No single outlier was responsible for the observed heterogeneity. In terms of study drop-out, both intervention and control groups showed aggregated proportions of 16%. Comparative analyses showed no significant difference in risk between AVATAR and control groups for both treatment and study drop-out (risk ratio of 1.01 in both cases).

**Table 5:** Meta-analytic drop-out results

Outcome	Timeframe/analysis	*k*	*N*_Experimental group, control group_	*I* ^2^ %	*I* ^2^ *p*-value	Effect size [95% CI]	Effect size *p*-value
Attrition (treatment drop-out)	Risk ratio	5	170 *v*. 166	49	0.10	1.01 [0.43–2.35]	0.98
	Proportion in experimental group	9	554	73	<0.001***	Proportion: 24% [0.17–0.33]	<0.001***
	Proportion in control group	5	166	59	0.005**	Proportion: 18 % [0.09–0.32]	<0.001***
Attrition (study drop-out)	Risk ratio	9	554 *v*. 424	0	0.45	Risk ratio: 1.01 [0.75–1.35]	0.96
	Proportion in experimental group	9	554	0	0.10	Proportion: 16% [0.14–0.20]	<0.001***
	Proportion in control group	9	424	15	0.13	16% [0.12–0.21]	<0.001***

*N,* sample size; *I* ^*2*^ , heterogeneity statistic. Negative effect sizes described lower scores in intervention groups whereas risk ratios above 1 describe an increased risk in intervention groups.

**p ≤ 0.01; ***p ≤ 0.001.

### Publication bias

The Egger’s test for AVH severity at post-treatment was significant (*p* = 0.009), indicating an asymmetric funnel plot.^59^ Examination of the corresponding funnel plot revealed the notable impact of the large effect size in Stefaniak et al.^66^ Trim-and-fill procedures revealed a corrected small effect size favouring AVATAR (Hedges’ *g*_*corrected*_ = −0.35, 95% CI −0.49 to −0.22, *p* < 0.001), with two studies estimated to be missing. The Egger’s test of AVH distress (*p* = 0.02) at post-treatment was significant. Examination of the corresponding funnel plot revealed the impact of the large effect sizes in Stefaniak et al^66^ and Percie du Sert et al.^65^ Trim-and-fill procedures revealed a corrected small effect size favouring AVATAR (Hedges’ *g*_*corrected*_ = −0.28, 95% CI −0.41 to −0.14, *p* < 0.001), with two studies estimated to be missing. Funnel plots and Egger’s tests statistics can be found in Supplementary Figs 7–10 and Supplementary Table 11, respectively.

## Discussion

The present study employed a systematic review and meta-analysis framework to investigate the efficacy, tolerability and acceptability of AVATAR for AVHs. Analyses showed preliminary evidence of the efficacy of AVATAR in decreasing AVH severity at post-treatment and short-term follow-up. This was robust for the effect of bias, and after correction for potential publication bias, although the effect was moderated by control group. Notably, the analyses were performed in persons with treatment-resistant and persistent AVHs, suggesting that AVATAR may address a critical treatment gap for previously refractory symptoms. The effect size observed (Hedges’ *g* = –0.40) was comparable to that of guideline-recommended psychological interventions, such as CBT (Hedges’ *g* = –0.34); however, this was in populations not specifically characterised as treatment-resistant.^21^ This illustrates the compelling potential of a theory-based intervention that harnesses digital technologies in a novel and effective way to treat AVHs. Medium- and long-term follow-ups (non-significant small effect sizes) were measured by only one study, limiting interpretability and certainty of evidence.

Analyses of effects on AVH frequency and distress showed significant small effects at post-treatment. They remained small and significant at short-term follow-up, and robust for the effect of bias (exception: distress at short-term follow-up) and potential publication bias. From a theoretical standpoint, the effects may align with potential mechanisms underlying reductions in AVH severity. The exposure to distressing avatar utterances and subsequent assertive strategies seem to have reduced both the frequency and distress of AVHs. Qualitative evidence has shown that although those affected by AVHs prioritise a decrease in the frequency of AVHs (i.e. total cessation), a decrease in AVH distress and disruption is more prioritised by service providers.^76^ These findings indicate that AVATAR may support shared goals between patients and caregivers, with the potential for mutually beneficial outcomes. Of note is that the duration of AVATAR generally consisted of fewer sessions (7–12) than a typical minimal dose of 16 sessions of a psychological intervention such as CBT,^9^ emphasising the efficiency of AVATAR. Nevertheless, AVATAR is comparatively resource-intensive and may be demanding to implement at scale, because of the requirements of software, hardware and training.^64^

Some concerns of publication bias were present in the AVH symptom analyses at post-treatment for AVH severity and distress. Funnel plots revealed the strong influence two small outlier studies with large effect sizes.^65,66^ This may point less toward the potential for missing studies and may instead reflect true heterogeneity of small studies. These are often able to direct more resources into treatment intensity, but are also more likely to be afflicted by methodological effects of bias.^77^ Furthermore, the outlier studies had waitlist control groups, which a recent meta-analysis has shown to have the smallest within-group symptom reductions in treatment-resistant schizophrenia,^78^ underlining the potential for inflated effects in these designs.

AVATAR therapy was generally well tolerated. Treatment-related adverse events and lasting symptom exacerbations were not common. However, two studies reported instances of anxiety occurring during early exposure to avatars,^65,69^ and a few cases requiring hospital admission attributable to treatment-related AVH symptom exacerbations.^69^ As with other exposure-based therapies, initial increases in anxiety are not unusual and typically diminish as the treatment progresses.^79^ Consistent with this, two included studies found that within-session anxiety significantly decreased over the course of the treatment.^26,27,65^ Additionally, small effect sizes favoured AVATAR in the reduction of anxiety symptoms at post-treatment and short-term follow-up. This further suggests that anxiety exacerbation is likely transient for the majority of participants. Studies also employed techniques to mitigate acute distress, such as offering a panic button or showing images of a calming beach.^25,69^ However, expanding upon these techniques to avoid overwhelming participants may be beneficial in AVATAR to minimise adverse events and attrition.

Treatment non-completion was observed in approximately a quarter of participants receiving AVATAR, which did not differ significantly in drop-out risk to control groups, supporting the general acceptability of the intervention. Notably, other exposure-based interventions report similar treatment drop-out rates (e.g. 28% for prolonged exposure in post-traumatic stress disorder^80^). The aggregated study drop-out rate of 16% across intervention and control conditions closely aligns with drop-out rates of CBT in schizophrenia spectrum disorders (14%^81^). This suggests that AVATAR is comparable to current recommended evidence-based treatments in terms of retention.

Positive symptoms improved compared to control groups, which is likely associated with the improvement in AVH symptoms. Effect sizes were comparable to those reported for CBT in treatment-resistant schizophrenia compared with TAU and supportive counselling (Hedges’ *g* = –0.31 and –0.19, respectively^82^). Total psychotic and negative symptoms did not show significant improvement, which may reflect the more specific focus on AVHs. In contrast, the small effect on anxiety symptoms at post-treatment and short-term follow-up makes the anxiolytic effect of exposure to distressing AVHs apparent. Significant results were not observed for social functioning and quality of life. Contrary to common impairment in schizophrenia spectrum disorders,^83^ functional outcomes were measured in few of the included studies, reflecting a common oversight in schizophrenia spectrum disorder research.^84^ Future trials should aim to consistently include functional outcome measures.

### Limitations

The results of this study should be considered alongside several limitations. As a meta-analysis of under ten studies, robustness of results is not assured, requiring additional high-quality, randomised controlled trials. Likewise, publication bias tests and meta-regression tests may have been underpowered, potentially missing significant effects.^77,85^ Another limiting factor concerns the length of measurement: only one study measured follow-ups later than 3 months post-intervention. Future studies are encouraged to perform longer follow-ups to assess the retention of effects. Although statistical heterogeneity was non-significant in the majority of analyses and often controlled by the removal of outliers, differences in treatment and follow-up durations, inclusion criteria, study designs, outcome measures and immersion were observed, potentially leading to clinical heterogeneity not assessed in these analyses. Future reviews should plan further subgroup and meta-regression analyses. Similarly, the question remains as to the comparative efficacy and tolerability of immersive virtual reality-based versus less immersive screen-based approaches, which could not be answered in this review and may be assessed with future direct comparison trials. Many included samples were small, had heterogeneous outcomes and were monocentric, with one study enrolling only 19 participants,^65^ potentially introducing bias into effect sizes.^86^ Future trials should be designed as large, multicentric trials with standardised outcome measures to allow for greater comparability between studies. Finally, trials were overwhelmingly conducted in Western, Educated, Industrial, Rich and Democratic (WEIRD) countries,^87^ limiting the generalisability of results for all populations reporting AVHs, especially considering the strong cultural aspects apparent in AVHs.^88,89^

In conclusion, AVATAR therapy showed efficacious and robust results for our primary outcome of the severity of AVH at post treatment, with moderate certainty of evidence. Effects were maintained into short-term follow-up, and AVH dimensions of both frequency and distress showed similar results. Efficacy profiles for other clinical and functional outcomes were mixed. These findings support the efficacy of AVATAR as a focused treatment for AVHs, although heterogeneity was apparent, and additional medium- and long-term evidence is required to assess the retention of effects.

## Supporting information

10.1192/bjo.2026.11014.sm001 Opper et al. supplementary material Opper et al. supplementary material

## Data Availability

Additional material is available in the online Supplementary Material. Data and scripts can be supplied upon reasonable request.

## Supplementary material

The supplementary material is available online at https://doi.org/10.1192/bjo.2026.11014
