# Supplementary material for: Audio Visual Assisted Therapy Aid for Refractory Auditory Hallucinations (AVATAR) therapy for voice hearers: systematic review and meta-analysis
Source: BJPsych Open. 2026 Apr 13;12(3):e104. doi: 10.1192/bjo.2026.11014 (PMC13107302; doi:10.1192/bjo.2026.11014)
Supplement: Opper et al. supplementary material [file S205647242611014Xsup001.docx]

**Supplementary Material**

[**Table S1:** PRISMA Abstracts Checklist 2](#_Toc210405132)

[**Table S2:** PRISMA Checklist 3](#_Toc210405133)

[**Table S3**. Search Strategy 7](#_Toc210405134)

[**Table S4**. Labels for Categorization of Inclusion and Exclusion of Records on *rayyan.ai* During Title and Abstract Screening 9](#_Toc210405135)

[**Table S5.** List of Excluded Studies Based on Full Text Screening 10](#_Toc210405136)

[**Table S6**. Additional Study Information 14](#_Toc210405137)

[**Table S7**. Additional Information Received from Study Authors 22](#_Toc210405138)

[**Table S8**. ROB-2 Ratings 23](#_Toc210405139)

[**Table S9**. Grading of Recommendations Assessment, Development and Evaluation (GRADE) 28](#_Toc210405140)

[**Table S10.** Included Measurements by Outcome and Study 30](#_Toc210405141)

[**Table S11**. Statistics for Publication Bias Tests 33](#_Toc210405142)

[**Figure S1**. PRISMA Screening Flow Diagram 34](#_Toc210405143)

[**Figure S2**. Forest Plots of AVH Frequency at Post-Treatment and Short-Term Follow-up 35](#_Toc210405144)

[**Figure S3**. Forest Plots of AVH Distress at Post-Treatment and Short-Term Follow-up 36](#_Toc210405145)

[**Figure S4**. Forest Plot of Psychotic Symptoms at Post-Treatment and Follow-up 37](#_Toc210405146)

[**Figure S5**. Forest Plot of Anxiety Symptoms, Depressive Symptoms, and Quality of Life at Post-Treatment and Follow-up 38](#_Toc210405147)

[**Figure S6**. Forest Plots of Treatment- and Study Dropout Proportion and Risk Ratio 39](#_Toc210405148)

[**Figure S7**. Funnel Plots for Voice-Related Outcomes at Post-Treatment and Follow-Up 40](#_Toc210405149)

[**Figure S8**. Funnel Plots for Psychotic Symptoms at Post-Treatment and Follow-Up 41](#_Toc210405150)

[**Figure S9**. Funnel Plots for Anxiety Symptoms, Depressive Symptoms, and Quality of Life at Post-Treatment and Follow-Up 42](#_Toc210405151)

[**Figure S10**. Funnel Plots for Treatment- and Study Dropout Proportion and Risk Ratio 43](#_Toc210405152)

[**References** 44](#_Toc210405153)

# **Table S1:** PRISMA Abstracts Checklist

| **Section and Topic** | **Item** | **Checklist item** | **Reported (Yes/No)** |
| --- | --- | --- | --- |
| **TITLE** | | |  |
| Title | 1 | Identify the report as a systematic review. | Yes |
| **BACKGROUND** | | |  |
| Objectives | 2 | Provide an explicit statement of the main objective(s) or question(s) the review addresses. | Yes |
| **METHODS** | | |  |
| Eligibility criteria | 3 | Specify the inclusion and exclusion criteria for the review. | Yes |
| Information sources | 4 | Specify the information sources (e.g. databases, registers) used to identify studies and the date when each was last searched. | Yes |
| Risk of bias | 5 | Specify the methods used to assess risk of bias in the included studies. | Yes |
| Synthesis of results | 6 | Specify the methods used to present and synthesise results. | Yes |
| **RESULTS** | | |  |
| Included studies | 7 | Give the total number of included studies and participants and summarise relevant characteristics of studies. | Yes |
| Synthesis of results | 8 | Present results for main outcomes, preferably indicating the number of included studies and participants for each. If meta-analysis was done, report the summary estimate and confidence/credible interval. If comparing groups, indicate the direction of the effect (i.e. which group is favoured). | Yes |
| **DISCUSSION** | | |  |
| Limitations of evidence | 9 | Provide a brief summary of the limitations of the evidence included in the review (e.g. study risk of bias, inconsistency and imprecision). | Yes |
| Interpretation | 10 | Provide a general interpretation of the results and important implications. | Yes |
| **OTHER** | | |  |
| Funding | 11 | Specify the primary source of funding for the review. | No funding |
| Registration | 12 | Provide the register name and registration number. | Yes |

*From.*  Page MJ, McKenzie JE, Bossuyt PM, Boutron I, Hoffmann TC, Mulrow CD, et al. The PRISMA 2020 statement: an updated guideline for reporting systematic reviews. BMJ 2021;372:n71. doi: 10.1136/bmj.n71.

#

# **Table S2:** PRISMA Checklist

| **Section and Topic** | **Item #** | **Checklist item** | **Location where item is reported** |
| --- | --- | --- | --- |
| **TITLE** | | |  |
| Title | 1 | Identify the report as a systematic review. | 1 |
| **ABSTRACT** | | |  |
| Abstract | 2 | See the PRISMA 2020 for Abstracts checklist. | See above |
| **INTRODUCTION** | | |  |
| Rationale | 3 | Describe the rationale for the review in the context of existing knowledge. | 4-6 |
| Objectives | 4 | Provide an explicit statement of the objective(s) or question(s) the review addresses. | 6 |
| **METHODS** | | |  |
| Eligibility criteria | 5 | Specify the inclusion and exclusion criteria for the review and how studies were grouped for the syntheses. | 8 |
| Information sources | 6 | Specify all databases, registers, websites, organisations, reference lists and other sources searched or consulted to identify studies. Specify the date when each source was last searched or consulted. | 7 |
| Search strategy | 7 | Present the full search strategies for all databases, registers and websites, including any filters and limits used. | Supplement Table S3 |
| Selection process | 8 | Specify the methods used to decide whether a study met the inclusion criteria of the review, including how many reviewers screened each record and each report retrieved, whether they worked independently, and if applicable, details of automation tools used in the process. | 7-9 |
| Data collection process | 9 | Specify the methods used to collect data from reports, including how many reviewers collected data from each report, whether they worked independently, any processes for obtaining or confirming data from study investigators, and if applicable, details of automation tools used in the process. | 8-9 |
| Data items | 10a | List and define all outcomes for which data were sought. Specify whether all results that were compatible with each outcome domain in each study were sought (e.g. for all measures, time points, analyses), and if not, the methods used to decide which results to collect. | 8-9 |
|  | 10b | List and define all other variables for which data were sought (e.g. participant and intervention characteristics, funding sources). Describe any assumptions made about any missing or unclear information. | Supplement Table S6 |
| Study risk of bias assessment | 11 | Specify the methods used to assess risk of bias in the included studies, including details of the tool(s) used, how many reviewers assessed each study and whether they worked independently, and if applicable, details of automation tools used in the process. | 10 |
| Effect measures | 12 | Specify for each outcome the effect measure(s) (e.g. risk ratio, mean difference) used in the synthesis or presentation of results. | 9 |
| Synthesis methods | 13a | Describe the processes used to decide which studies were eligible for each synthesis (e.g. tabulating the study intervention characteristics and comparing against the planned groups for each synthesis (item #5)). | 9-10 |
|  | 13b | Describe any methods required to prepare the data for presentation or synthesis, such as handling of missing summary statistics, or data conversions. | 9 |
|  | 13c | Describe any methods used to tabulate or visually display results of individual studies and syntheses. | 9 |
|  | 13d | Describe any methods used to synthesize results and provide a rationale for the choice(s). If meta-analysis was performed, describe the model(s), method(s) to identify the presence and extent of statistical heterogeneity, and software package(s) used. | 9-10 |
|  | 13e | Describe any methods used to explore possible causes of heterogeneity among study results (e.g. subgroup analysis, meta-regression). | 9-10 |
|  | 13f | Describe any sensitivity analyses conducted to assess robustness of the synthesized results. | 9-10 |
| Reporting bias assessment | 14 | Describe any methods used to assess risk of bias due to missing results in a synthesis (arising from reporting biases). | 10 |
| Certainty assessment | 15 | Describe any methods used to assess certainty (or confidence) in the body of evidence for an outcome. | 10 |
| RESULTS | | |  |
| Study selection | 16a | Describe the results of the search and selection process, from the number of records identified in the search to the number of studies included in the review, ideally using a flow diagram. | Supplement Figure S1 |
|  | 16b | Cite studies that might appear to meet the inclusion criteria, but which were excluded, and explain why they were excluded. | Supplement Table S4-5 |
| Study characteristics | 17 | Cite each included study and present its characteristics. | 12 |
| Risk of bias in studies | 18 | Present assessments of risk of bias for each included study. | 14 |
| Results of individual studies | 19 | For all outcomes, present, for each study: (a) summary statistics for each group (where appropriate) and (b) an effect estimate and its precision (e.g. confidence/credible interval), ideally using structured tables or plots. | Tables 2-4 |
| Results of syntheses | 20a | For each synthesis, briefly summarise the characteristics and risk of bias among contributing studies. | Supplement Tables S2-6 |
|  | 20b | Present results of all statistical syntheses conducted. If meta-analysis was done, present for each the summary estimate and its precision (e.g. confidence/credible interval) and measures of statistical heterogeneity. If comparing groups, describe the direction of the effect. | 15-21 |
|  | 20c | Present results of all investigations of possible causes of heterogeneity among study results. | 15-21 |
|  | 20d | Present results of all sensitivity analyses conducted to assess the robustness of the synthesized results. | 15-21 |
| Reporting biases | 21 | Present assessments of risk of bias due to missing results (arising from reporting biases) for each synthesis assessed. | 21 |
| Certainty of evidence | 22 | Present assessments of certainty (or confidence) in the body of evidence for each outcome assessed. | 15/Supplement Table S9 |
| **DISCUSSION** | | |  |
| Discussion | 23a | Provide a general interpretation of the results in the context of other evidence. | 21-24 |
|  | 23b | Discuss any limitations of the evidence included in the review. | 24-25 |
|  | 23c | Discuss any limitations of the review processes used. | 24-25 |
|  | 23d | Discuss implications of the results for practice, policy, and future research. | 23, 25 |
| **OTHER INFORMATION** | | |  |
| Registration and protocol | 24a | Provide registration information for the review, including register name and registration number, or state that the review was not registered. | 3 |
|  | 24b | Indicate where the review protocol can be accessed, or state that a protocol was not prepared. | None |
|  | 24c | Describe and explain any amendments to information provided at registration or in the protocol. | 10 |
| Support | 25 | Describe sources of financial or non-financial support for the review, and the role of the funders or sponsors in the review. | 26 |
| Competing interests | 26 | Declare any competing interests of review authors. | 26 |
| Availability of data, code and other materials | 27 | Report which of the following are publicly available and where they can be found: template data collection forms; data extracted from included studies; data used for all analyses; analytic code; any other materials used in the review. | 26 |

*From:*  Page MJ, McKenzie JE, Bossuyt PM, Boutron I, Hoffmann TC, Mulrow CD, et al. The PRISMA 2020 statement: an updated guideline for reporting systematic reviews. BMJ 2021;372:n71. doi: 10.1136/bmj.n71

# **Table S3**. Search Strategy

| **Database** | **Search string** | **Date** | ***k* results** |
| --- | --- | --- | --- |
| **PubMed** | ("Virtual Reality"[Mesh] OR "Therapy, Computer-Assisted"[Mesh] OR "Avatar"[tiab] OR "Virtual Realit*"[tiab] OR "VR"[tiab] OR "digital therap*"[tiab] OR "technology assisted therap*"[tiab]) AND ("Schizophrenia Spectrum and Other Psychotic Disorders"[Mesh] OR "schizophreni*"[tiab] OR "psychot*"[tiab] OR “psychosis”[tiab]) AND ("auditory hallucination*"[tiab] OR "Hallucinations"[Mesh] OR "voice*"[tiab]) | 21.03.25 | 62 |
| **PsycInfo** | (DE "Virtual Reality" OR DE "Augmented Reality" OR DE "Computer Assisted Therapy" OR DE "Avatars" OR (TI("Avatar" OR Virtual Realit* OR "VR" OR digital therap* OR technology assisted therap* OR computer assisted therap*) OR AB("Avatar" OR Virtual Realit* OR "VR" OR digital therap* OR technology assisted therap* OR computer assisted therap*))) AND (DE "Psychosis" OR DE "Affective Psychosis" OR DE "Alcohol Induced Psychotic Disorders" OR DE "Brief Psychotic Disorder" OR DE "Capgras Syndrome" OR DE "Childhood Onset Psychosis" OR DE "Chronic Psychosis" OR DE "Delusional Disorder" OR DE "Experimental Psychosis" OR DE "Hallucinosis" OR DE "Paranoid Psychosis" OR DE "Postpartum Psychosis" OR DE "Reactive Psychosis" OR DE "Schizophrenia" OR DE "Substance Induced Psychotic Disorders" OR (TI("schizophreni*" OR "psychot*" OR “psychosis”) OR AB("schizophreni*" OR "psychot*" OR “psychosis”))) AND (DE "Hallucinations" OR DE "Auditory Hallucinations" OR AB(auditory hallucination* OR "voice*") OR TI(auditory hallucination* OR "voice*")) | 21.03.25 | 73 |
| **Web of Science** | #1 (TI=("schizophreni*" OR "psychot*" OR “psychosis”)) OR (AB=("schizophreni*" OR "psychot*" OR “psychosis”))  #2 (TI=("Avatar" OR Virtual Realit* OR "VR" OR digital therap* OR technology assisted therap* OR computer assisted therap*)) OR (AB=("Avatar" OR Virtual Realit* OR "VR" OR digital therap* OR technology assisted therap* OR computer assisted therap*))   #3 (TI=("Hallucination*" OR "voice*")) OR (AB=("Hallucination*" OR "voice*"))  #4 #1 AND #2 AND #3 | 21.03.25 | 113 |
| **EMBASE** | #1 exp psychosis/ or ('schizophreni*' or 'psychot*' or 'psychosis').ti,ab.  #2 exp virtual reality/ or ('Avatar' or 'Virtual Realit*' or 'VR' or 'digital therap*' or 'technology assisted therap*' OR  ‘computer assisted therap*’).ti,ab.  #3 ('Hallucination*' or 'voice*').ti,ab.  #4 #1 AND #2 AND #3 | 21.03.25 | 149 |
| **ClinicalTrials.gov** | Other terms: (schizophrenia OR psychotic OR psychosis) AND (Avatar OR Virtual Reality OR VR OR digital therapy OR technology assisted therapy OR computer assisted therapy) AND (Hallucination OR voice) | 21.03.25 | 62 |
| **CENTRAL** | #1: MeSH descriptor: [Schizophrenia Spectrum and Other Psychotic Disorders] explode all trees  #2: (schizophreni* OR psychot* OR “psychosis”):ti,ab,kw  #3: MeSH descriptor: [Virtual Reality] explode all trees  #4: MeSH descriptor: [Therapy, Computer-Assisted] explode all trees  #5: MeSH descriptor: [avatar] explode all trees  #6: ("Avatar" OR (Virtual Realit*) OR "VR" OR (digital therap*) OR (technology assisted therap*) OR (computer assisted therap*)):ti,ab,kw  #7: (Hallucination* OR voice*):ti,ab,kw  #8: MeSH descriptor: [Hallucinations] explode all trees  #9: (#1 OR #2) AND (#3 OR #4 OR #5 OR #6) AND (#7 OR #8) | 21.03.25 | 95 |
| **ISRCTN** | ("schizophreni*" OR "psychot*" OR “psychosis”) AND ("Avatar" OR "Virtual Realit*" OR "VR" OR "digital therap*" OR "technology assisted therap*" OR "computer assisted therap*") AND ("hallucination*" OR "voice*") | 21.03.25 | 2 |
| **Duplicates** |  |  | 211 |

# **Table S4**. Labels for Categorization of Inclusion and Exclusion of Records on *rayyan.ai* During Title and Abstract Screening

| **Label** | **Description** | **Key (Rayyan)** | **Number of Cases** |
| --- | --- | --- | --- |
| **Ineligible intervention** | Did not consider AVATAR or equivalent intervention for auditory verbal hallucinations | A | 226 |
| **Background article** | Non study descriptive background article | B | 2 |
| **Ineligible study design** | Non (n)RCT (e.g. Case series) | S | 41 |
| **Ineligible population** | Did not include samples of participants (16+ years) with auditory verbal hallucinations and majority diagnosis with psychotic disorder | P | 5 |
| **Ineligible outcome** | Did not include any of the pre-registered outcomes | O | 0 |
| **Outdated version** | Record is outdated; an updated version of the paper should be found | V | 0 |
| **Retraction** | Record has been retracted. | R | 0 |

*Note*. Labels are listed in hierarchical order of the screening process.

#

# **Table S5.** List of Excluded Studies Based on Full Text Screening

| **Authors** | | **Year** | **Title** | **Exclusion reason** |
| --- | --- | --- | --- | --- |
| Craig, Tom K. J.; Rus-Calafell, Mar; Ward, Thomas; Leff, Julian P.; Huckvale, Mark; Howarth, Elizabeth; Emsley, Richard; Garety, Philippa A. | 2018 | | AVATAR therapy for auditory verbal hallucinations in people with psychosis: A single-blind, randomised controlled trial. | outdated version: correction available |
| Garety PA; Edwards CJ; Jafari H; Emsley R; Huckvale M; Rus-Calafell M; Fornells-Ambrojo M; Gumley A; Haddock G; Bucci S; McLeod HJ; McDonnell J; Clancy M; Fitzsimmons M; Ball H; Montague A; Xanidis N; Hardy A; Craig TKJ; Ward T | 2024 | | Digital AVATAR therapy for distressing voices in psychosis: the phase 2/3 AVATAR2 trial. | outdated version: correction available |
| Dellazizzo, L; Potvin, S; O'Connor, K; Dumais, A | 2018 | | A randomized controlled trial comparing virtual reality therapy to cognitive behavioral therapy in schizophrenia with treatment refractory hallucinations: preliminary results | outdated version: correction available |
| Craig T.; Garety P.; Ward T.; Rus-Calafell M.; Williams G.; Huckvale M.; Leff J. | 2014 | | Computer assisted therapy for auditory hallucinations: The avatar clinical trial | conference proceeding (congruence with already published trial) |
| Leff J.; Williams G.; Huckvale M.A.; Arbuthnot M.; Leff A.P. | 2013 | | Computer-assisted therapy for medication-resistant auditory hallucinations: Proof-of-concept study | outdated version: correction available |
| Dellazizzo, Laura; Percie du Sert, Olivier; Phraxayavong, Kingsada; Potvin, Stéphane; O'Connor, Kieron; Dumais, Alexandre | 2018 | | Exploration of the dialogue components in Avatar Therapy for schizophrenia patients with refractory auditory hallucinations: A content analysis. | secondary article |
| Rus-Calafell, Mar; Ehrbar, Nils; Ward, Thomas; Edwards, Clementine; Huckvale, Mark; Walke, Jennifer; Garety, Philippa; Craig, Tom | 2022 | | Participants’ experiences of AVATAR therapy for distressing voices: A thematic qualitative evaluation. | secondary article |
| Craig, Tom; Ward, Tom; Rus-Calafell, Mar | 2016 | | AVATAR therapy for refractory auditory hallucinations. | conference proceeding (congruence with alreadypublished trial) |
| NCT06628323 | 2024 | | The Neuro-VR Study: A Pilot Study Investigating an Innovative Virtual Reality-based Intervention Employing Neurofeedback to Increase Tolerability and Therapy Efficacy in Psychosis | non-completed trial – Authors contacted – unfinished |
| University of Barcelona; Parc Sanitari Sant Joan de Déu; Fundació Sant Joan de Déu; Uniwersytet Medyczny w Łodzi | 2024 | | VR-based Avatar Therapy for Treatment of Auditory Hallucinations in Patients With Schizophrenia 2023-SUD-3446 | non-completed trial – Authors contacted – unfinished |
| ZonMw: The Netherlands Organisation for Health Research and Development | 2023 | | Virtual Reality Therapy for Voice Hearing (VR-VOICES): a Randomized Controlled Trial | non-completed trial – unfinished according to ClinicalTrials.gov |
| University of Copenhagen; University of Manchester; University of Melbourne; Monash University; Perth Voices Clinic; University of the Sunshine Coast; University of Toronto | 2023 | | Remotely Delivered Avatar-mediated Therapy Versus Cognitive Behavioural Therapy for Persisting Auditory Hallucinations: Randomised Controlled Superiority Trial | non-completed trial – authors contacted – unfinished |
| Mental Health Services in the Region of Southern Denmark; Mental Health Services in the North Denmark Region | 2020 | | The CHALLENGE-trial: the Effects of a Virtual Reality-assisted Exposure Therapy for Persistent Auditory Hallucinations Versus Supportive Counselling in People With Psychosis | outdated version: registration |
| Psychiatric University Hospital, Zurich | 2019 | | Virtual Reality Avatar Therapy for People Hearing Voices | non-completed trial – authors contacted – unfinished |
| Canadian Institutes of Health Research (CIHR) | 2019 | | A Randomized Controlled Trial Comparing Avatar Therapy to Cognitive Behavioral Therapy in Schizophrenia With Treatment Refractory Hallucinations | outdated version: registration |
| Otsuka Canada Pharmaceutical Inc. | 2018 | | A Pilot Randomized Controlled Study to Compare Avatar Therapy and Cognitive Behavioral Therapy in Patients With Treatment-resistant Schizophrenia. | outdated version: registration |
| Centre de Recherche de l'Institut Universitaire en santé Mentale de Montréal | 2017 | | Emotion Regulation Using Virtual Reality in Schizophrenia: a Clinical and Mechanistic Trial of Avatar Therapy for Refractory Auditory Hallucinations | outdated version: registration |
| Marotti J; Saunders R; Montague A; Fornells-Ambrojo M | 2025 | | The role of trauma, attachment, and voice-hearer's appraisals: a latent profile analysis in the AVATAR2 trial. | secondary article |
| Hudon A; Quilliam S; Phraxayavong K; Potvin S; Dumais A | 2024 | | Exploring Therapeutic Outcomes Through Dyadic Interactions: The Role of Patient-Avatar Dynamics in Avatar Therapy. | secondary article |
| Smith J | 2024 | | Daily briefing: Avatar therapy helps people with psychosis stand up to the voices in their heads. | secondary article |
| Hudon A; Beaudoin M; Phraxayavong K; Potvin S; Dumais A | 2023 | | Enhancing Predictive Power: Integrating a Linear Support Vector Classifier with Logistic Regression for Patient Outcome Prognosis in Virtual Reality Therapy for Treatment-Resistant Schizophrenia. | secondary article |
| Hudon A; Lammatteo V; Rodrigues-Coutlée S; Dellazizzo L; Giguère S; Phraxayavong K; Potvin S; Dumais A | 2023 | | Exploration of the role of emotional expression of treatment-resistant schizophrenia patients having followed virtual reality therapy: a content analysis. | secondary article |
| Hudon A; Beaudoin M; Phraxayavong K; Potvin S; Dumais A | 2023 | | Unsupervised Machine Learning Driven Analysis of Verbatims of Treatment-Resistant Schizophrenia Patients Having Followed Avatar Therapy. | secondary article |
| Dellazizzo L; Potvin S; Phraxayavong K; Giguère S; Hamidi LN; Dumais A | 2021 | | [Improving Quality of Life in Patients with Refractory Schizophrenia After Virtual Reality-assisted Therapy: A Content Analysis]. | secondary article |
| Garety P; Edwards CJ; Ward T; Emsley R; Huckvale M; McCrone P; Rus-Calafell M; Fornells-Ambrojo M; Gumley A; Haddock G; Bucci S; McLeod H; Hardy A; Peters E; Myin-Germeys I; Craig T | 2021 | | Optimising AVATAR therapy for people who hear distressing voices: study protocol for the AVATAR2 multi-centre randomised controlled trial. | outdated version: protocol |
| Brander M; Egger ST; Hürlimann N; Seifritz E; Sumner RW; Vetter S; Magnenat S | 2021 | | Virtual Reality Human-Human Interface to Deliver Psychotherapy to People Experiencing Auditory Verbal Hallucinations: Development and Usability Study. | incorrect population |
| Craig T; Ward T; Rus-Calafell M | 2016 | | AVATAR Therapy for Refractory Auditory Hallucinations. | outdated version: protocol |
| Ward, T; Jamieson-Craig, T; Garety, PA; Rus-Calafell, M; McCrone, P; Emsley, R; Williams, G; Huckvale, M; Leff, J | 2014 | | A randomized clinical trial of a novel audio-visual assisted therapy aid for refractory auditory hallucinations (AVATAR therapy) | conference proceeding – congruence with already published trial |
| Craig, TK; Rus-Calafell, M; Ward, T; Fornells-Ambrojo, M; McCrone, P; Emsley, R; Garety, P | 2015 | | The effects of an Audio Visual Assisted Therapy Aid for Refractory auditory hallucinations (AVATAR therapy): study protocol for a randomised controlled trial | outdated version: protocol |
| ISRCTN35980117 | 2023 | | AVATAR_VRSocial therapy for auditory verbal hallucinations in early psychosis | non-completed trial – authors contacted – unfinished |
| ISRCTN55682735 | 2020 | | Optimising AVATAR therapy for distressing voices | outdated version: registration |
| Craig, T; Garety, P; Ward, T; Edwards, C; Rus-Calafell, M; Huckvale, M; Emsley, R | 2022 | | The UK AVATAR 1 and 2 Trials for People with Distressing Voices - Findings and Learning from AVATAR1, and AVATAR2 Developments in Theory and Therapy | secondary article |
| Dzafic, I; Spark, J; Bell, I; Wood, S; Lavoie, S; Whitford, T; Yuen, HP; Thomas, N; Thompson, A; Alvarez-Jimenez, M | 2023 | | PAPER: the hybrid study: integrating neurofeedback, virtual reality, and cognitive behaviour therapy for the treatment of hearing voices | conference proceeding – non RCT |
| NCT03585127 | 2018 | | Avatar Therapy in Comparison to Cognitive Behavioral Therapy for Treatment-resistant Schizophrenia | outdated version: registration |
| NCT04054778 | 2019 | | Comparaison of Avatar Therapy to Cognitive Behavioral Therapy in Schizophrenia With Treatment Refractory Hallucinations | outdated version: registration |
| Glenthoj, LB | 2023 | | PAPER: using immersive virtual reality in treating paranoia and auditory hallucinations | conference proceeding – non RCT |
| NCT06628323 | 2024 | | Neuro-VR: augmenting a Virtual Reality-based Therapy with Neurofeedback for Auditory Hallucinations | non-completed trial – authors contacted – unfinished |
| ChiCTR1900027254 | 2019 | | Virtual reality-assisted therapy for auditory verbal hallucinations: a multicenter randomized controlled clinical trial | outdated version: registration |
| NCT05982158 | 2023 | | Avatar-mediated Therapy Versus Cognitive Behavioural Therapy for Persisting Experiences of Hearing Voices | non-completed trial – authors contacted – unfinished |
| NCT04661163 | 2020 | | CHALLENGE. A Randomised Clinical Trial Examining Virtual Reality Therapy | outdated version: registration |
| Glenthoj, L; Smith, L; Mariegaard, L; Due, AS; Christensen, A; Christensen, M; Vernal, D; Jeppesen, U; Nordentoft, M | 2022 | | CHALLENGE and Face Your Fears: virtual Reality Treatment for Auditory Hallucinations and Paranoid Ideations | conference proceeding |
| NCT03148639 | 2017 | | Virtual Reality Therapy for Treatment-resistant Auditory Hallucinations in Schizophrenia | outdated version: registration |
| NL-OMON52102 | 2022 | | Virtual reality therapy for voice hearing: a randomized controlled trial | non-completed trial – authors contacted – unfinished |
| IRCT20220226054121N1 | 2022 | | AVATAR therapy in refractory auditory hallucinations | trial registration – no reply |
| ChiCTR2100053045 | 2021 | | Psychotherapy of auditory hallucinations in schizophrenia under digital Avarta | trial registration – no reply |
| Liang N.; Li X.; Guo X.; Liu S.; Liu Y.; Zhao W.; Wen Y.; Li Y.; Li F.; Wu H.; Li J.; Li Q.; Guo J.; Xu Y. | 2023 | | P50 sensory gating as a neurophysiological correlate of symptomatic improvement by virtual reality-based computer avatar therapy system in patients with auditory verbal hallucinations: A pilot study | secondary article |
| Hudon A.; Couture J.; Dellazizzo L.; Beaudoin M.; Phraxayavong K.; Potvin S.; Dumais A. | 2023 | | Dyadic Interactions of Treatment-Resistant Schizophrenia Patients Having Followed Virtual Reality Therapy: A Content Analysis | secondary article |
| Beaudoin M.; Potvin S.; Phraxayavong K.; Dumais A. | 2023 | | Changes in quality of life in treatment-resistant schizophrenia patients undergoing VR-assisted therapy for auditory verbal hallucinations: A content analysis | secondary article |
| Beaudoin M.; Potvin S.; Dumais A. | 2023 | | Comparison of VR-Assisted Therapy to Cognitive- Behavioral Therapy in the treatment of verbal hallucinations in patient with treatment-resistant schizophrenia | non-completed trial – according to ClinicalTrials.gov |
| Ster A.C.; Emsley R.; Landau S. | 2023 | | COMBINING NON-ADHERENCE AND MEDIATION IN A UNIFIED CAUSAL ANALYSIS: A METHODOLOGICAL REVIEW AND APPLICATION TO THE AVATAR TRIAL | secondary article |
| Hudon A.; Leveille N.; Sanchez-Schicharew K.; Dellazizzo L.; Phraxayavong K.; Dumais A. | 2022 | | The impacts of the COVID-19 pandemic on treatment-resistant schizophrenia patients having followed virtual reality therapy or cognitive behavioural therapy: a content analysis | secondary article |
| Hudon A.; Beaudoin M.; Phraxayavong K.; Dellazizzo L.; Potvin S.; Dumais A. | 2022 | | Implementation of a machine learning algorithm for automated thematic annotations in avatar: A linear support vector classifier approach | secondary article |
| O'Brien C.; Rus-Calafell M.; Craig T.K.; Garety P.; Ward T.; Lister R.; Fornells-Ambrojo M. | 2021 | | Relating behaviours and therapeutic actions during AVATAR therapy dialogue: An observational study | secondary article |
| Ward T.; Lister R.; Fornells-Ambrojo M.; Rus-Calafell M.; Edwards C.J.; O'Brien C.; Craig T.K.; Garety P. | 2021 | | The role of characterisation in everyday voice engagement and AVATAR therapy dialogue | secondary article |
| Ward T.; Rus-Calafell M.; Ramadhan Z.; Soumelidou O.; Fornells-Ambrojo M.; Garety P.; Craig T.K.J. | 2020 | | AVATAR therapy for distressing voices: A comprehensive account of therapeutic targets | secondary article |
| Dellazizzo L.; Potvin S.; Phraxayavong K.; Dumais A. | 2020 | | Exploring the benefits of virtual reality-assisted therapy following cognitive-behavioral therapy for auditory hallucinations in patients with treatment-resistant Schizophrenia: A proof of concept | no control group |
| Craig T. | 2020 | | Virtual reality and the avatar | secondary article |
| Craig T.K.J. | 2019 | | AVATAR therapy: a promising new approach for persistent distressing voices | secondary article |
| Garety P.; Craig T.; Rus-Callafel M.; Ward T.; Emsley R.; Huckvale M. | 2019 | | AVATAR therapy for distressing voices | secondary article |
| Garety P. | 2019 | | Talking to your voices might help: the use of Avatars in the treatment of psychosis | secondary article |
|  | 2018 | | Corrections: AVATAR therapy for auditory verbal hallucinations in people with psychosis: a single-blind, randomised controlled trial (The Lancet Psychiatry (2018) 5(1) (31-40) (S2215036617304273) (10.1016/S2215-0366(17)30427-3))      A1  - Anonymous. | secondary article |
| Rus-Calafell M.; Ward T.; Garety P.; Craig T. | 2016 | | Examining the role of presence in the AVATAR therapy | secondary article |
| Rus-Calafell M.; Ward T.; Garety P.; Craig T.K.J. | 2016 | | Examining the role of presence in the AVATAR therapy | secondary article |

# **Table S6**. Additional Study Information

| **Study** | **Mean age (*SD*)** | **Setting** | **Inclusion criteria** | **Exclusion criteria** | **AVATAR Protocol Info** | **Control Protocol Info** | **Diagnoses Distributions** | **Funding Information** | |
| --- | --- | --- | --- | --- | --- | --- | --- | --- | --- |
| Craig et al. 2018^1^ | 42.7 (10.7) | Outpatient | Distressing auditory verbal hallucinations for at least 12 months; diagnosis of schizophrenia spectrum disorder or affective disorder with psychotic symptoms (ICD-10); currently patient of NHS psychiatric services; age >18 years; English language ability; unresponsive or only partially responsive to previous antipsychotic medication | Unable to give informed written consent; currently receiving psychological therapy for psychosis or attending hearing voices groups; refusing medication; diagnosis of organic brain disease, learning disability, or primary substance dependency; heard voices in a language not spoken by therapists | 6 sessions of screen-based AVATAR + TAU:  Sessions 1-3: Creation of avatar for main source of voice;  assessment of voice(s) and  content; 10-15 mins per session exposure to avatar with typical voice content  while therapist encourages  assertive responding  Sessions 4-6:  avatar concedes and acknowledges qualities of participant  Recorded dialogue provided to participant with instructions to listen to recording at home | 6 sessions supportive counselling + TAU; empathic, non-directive therapeutic relationship; manual- based, face-to-face supportive counselling approach of SoCRATES Trial Group delivered by graduate assistant psychologists  with experience with psychosis^2^; trained and supervised throughout; topics: quality of life, identity or belonging, past trauma,  resources and qualities; participants recorded weekly positive message to listen back to during the week | 77% paranoid schizophrenia; 11% schizoaffective disorder; 5% bipolar disorder; 5% unspecific psychosis; 3% depression with psychotic symptoms | Wellcome Trust; (FWBC-AVATAR 098272/z/12/z);  National Institute for  Health Research (NIHR) Biomedical Research Centre at South London  and Maudsley NHS Foundation Trust; King’s College London. | |
| **Study** | **Mean age (*SD*)** | **Setting** | **Inclusion criteria** | **Exclusion criteria** | **AVATAR Protocol Info** | **Control Protocol Info** | **Diagnoses Distributions** | **Funding Information** |  |
| Dellazizzo et al. 2021^3^ | 42.5 (12.7) | Outpatient | Diagnosis of schizophrenia  or schizoaffective disorder with persistent AVHs; failure to respond to  two or more antipsychotic trials | No primary diagnosis of schizophrenia or schizoaffective disorder; not hearing distressing voices, reporting voices not speaking in the therapists’ primary language; in denial of voices; substance use problems; not stabilized with treatment), having received another psychological treatment; being under curatorship | 9-weekly VR AVATAR sessions + TAU: pre-immersion and objectives; immersion and dialog with avatar; post-immersion with debrief of feelings  Session 1: Creation of avatar for main source of voices; assessment of AVHs;  immersion in VR in first person perspective in a dark room; therapist in other room, conversed as themselves or as avatar;  avatar also employed emotional facial expressions  Sessions 2–4: Exposure to avatar to enhance emotional regulation and assertiveness.  Session 5: targeted self-esteem, enabling self-expression to express  Ending sessions: voices became less abusive/more supportive; application of previously learned tools | 9 sessions CBT + TAU: manualized individual and weekly sessions; administered by licensed psychologist trained in CBT; derived and adapted from current evidence-based treatments for AVH; succession of learning modules and suggested task assignments.  Session 1: history of voices  Sessions 2-3: assessing and learning about AVHs; voices as triggers rather than beliefs; voice journals as assignments, in order to reflect on positive symptoms and triggers.  Session 4: metacognition; attributional mechanisms and voice journal to detect beliefs  Sessions 5-6: reinterpreting situations with the use of vignettes.  Sessions 7-8: mindfulness exercises, asking for feedback and learning to observe; voice journal, with alternative explanations to beliefs about hallucinations.  Session 9: End of intervention relapse prevention. | 77% schizophrenia; 23% schizoaffective disorder | Pinel Foundation, Services et Recherches Psychiatriques AD, Levesque Foundation and Otsuka Canada Pharmaceuticals Inc. |  |
| **Study** | **Mean age (*SD*)** | **Setting** | **Inclusion criteria** | **Exclusion criteria** | **AVATAR Protocol Info** | **Control Protocol Info** | **Diagnoses Distributions** | **Funding Information** |  |
| Garety et al. 2024^4^ | 39.61 (13.26) | Outpatient | Aged 18 years or over; under the care of a specialist mental health team; current frequent and distressing voices (score of  at least one on each of the intensity of distress and frequency items  of the PSYRATS-AH), persisting for at least 6 months  and spoken in English; speak and read English to a sufficient level  to provide consent and complete the assessment procedures;  clinical diagnosis of schizophrenia spectrum disorder or affective disorder with psychotic symptoms (ICD-10) through clinical records and consultation with clinical team | Primary diagnosis of substance dis- order, personality disorder or learning disability; lacking capacity to consent; profound visual or hearing impairment or insufficient comprehension of English to be able to engage in assessment or treatment; currently undertaking individual psychological treatment for voices; currently experiencing an acute mental health crisis. | Screen based AVATAR Brief (6 sessions) and Extended (12 sessions) + TAU;  AVATAR Brief:  Initial clinical assessment session: creation of the avatar; exposure to verbatim voice content) focus on increasing power, control, and self-esteem.  AVATAR extended: phases of AVATAR brief; phase 2 develops understanding of voice(s) within context of life and relationship history, contribution of previous traumatic experience; dialogues targeting wider topics  Sessions with three parts each: (1) pre-dialogue discussion with review of previous week; discussion of relevant themes for dialogue (2) active avatar dialogue with recording; (3) post-dialogue debrief with comment on strengths shown by participant, emerging content, and encouragement | TAU with antipsychotic medication, contact with mental health worker and outpatient psychiatric appointments; no alteration of pharmacological or psychosocial treatment decisions | 43.8% schizophrenia; 0.9% Persistent delusional disorders; 1.2% Acute and transient psychotic disorders; 0.3% Induced delusional disorder; 7.8% schizoaffective disorder; 2.3% Other nonorganic psychotic disorders; 31% Unspecified nonorganic psychosis; 2.3% Bipolar affective disorder; 9.6% Severe depressive episode with psychotic symptoms | Wellcome grant no. 215471/Z/19/Z. |  |
| **Study** | **Mean age (*SD*)** | **Setting** | **Inclusion criteria** | **Exclusion criteria** | **AVATAR Protocol Info** | **Control Protocol Info** | **Diagnoses Distributions** | **Funding Information** |  |
| Leff et al. 2013^5^ | 38.88 (16.70) | Outpatient | Hearing persecutory voices for at least 6 months, no adequate response to antipsychotic medication; age between 14 and 75 | Organic brain disease; substance misuse | 6 sessions screen-based AVATAR + TAU  Session 1: creation of avatar  Following sessions:  dialogue with avatar with therapist encouragement to stand up to avatar; therapist controlled the avatar so progressively came under participants’ control and became less abusive and more helpful to reintegrate the exteriorized component of psyche; recording of each session for patients to use outside of sessions to reinforce control; Therapy could be halted by pressing a button causing avatar to disappear from monitor, to be replaced by scene of a tropical beach accompanied by soothing music | TAU + WL: ongoing antipsychotic medication and supervision by referring psychiatrist | 100% schizophrenia | National Institute for Health and Care Research (NIHR), Wellcome Trust; King’s Clinical Research Facility, NIHR Maudsley Biomedical Research Centre and Maudsley NHS Foundation Trust; King’s College London (P.A.G. and R.E., no. NIHR203318) ;Manchester Biomedical Research Centre (S.B. and G.H., no. NIHR203308). |  |
| **Study** | **Mean age (*SD*)** | **Setting** | **Inclusion criteria** | **Exclusion criteria** | **AVATAR Protocol Info** | **Control Protocol Info** | **Diagnoses Distributions** | **Funding Information** |  |
| Liang et al. 2022^6^ | Reported per group:  CATS: 25.3 (5.3)  CBT: 26.5 (6.8) | Outpatient | Suffered AVHs for at least 12 months and diagnosed with schizophrenia; medication resistance without remission of AVHs, despite two adequate  6-week trials of at least two antipsychotic medications, including one  atypical antipsychotic medication); no dose change in antipsychotics  drugs in the past 3 months; hearing voices in a language  spoken by Chinese; aged 19 years or older | Receiving psychological therapy and other treatments (electroconvulsive therapy, transcranial magnetic stimulation, magnetic seizure therapy, or transcranial direct current stimulation) within the past year; alcohol or substance use disorder; organic brain disease. | 7–9 weekly sessions of motion capture VR AVATAR + TAU; 10-15 min. exposure to avatar per session:  Session 1: avatar creation  Session 2-3: preparation for exposure; exposure to negative content; encouragement to contradict and learn emotional regulation and assertiveness;  Sessions 3-4; coping strategies; focus on reducing anxiety and gaining power; replacing counter-attack attitude with self-affirmation or ignoring;  Sessions 5-6: self-esteem reinforced with qualities provided by friends and family in dialogue  Sessions 7-8: meaning of voice in life; reducing sense of voices as omnipotent, weaken repetitiveness with voice or reconcile contradictory relationship; improve self-confidence, resilience, and mental flexibility  Session 9: relapse prevention  Audio recorded each session and provided to hear when the voices reappeared | CBT + TAU (7–9 sessions) licensed psychologist informed by evidence-based CBT approaches for AVHs  Session 1: introduction of therapy, self-assessment of AVHs; characteristics; distress level; identification of goals; daily voices log;  Sessions 2–3: cognitive models for voices, strategies used to cope; exploring and strengthening new ways to manage voices;  Sessions 3–4: identifying negative belief formation, maintenance, and impact; searching for disconfirming evidence.  Sessions 5–6: re-evaluating beliefs based on the disconfirming evidence; alternative explanatory models  Sessions 7–8: mindfulness and; mindful relation; decreasing emotional and behavioral resistant responses; mindfulness daily at home using supplied 5- or 10-minute recording  Session 9: relapse prevention and maintaining gains | 100% Schizophrenia | National Natural Science Foundation of China (81971601 and 81701326); National Key Research and Development Program of China (2016YFC1307004), Support Program of the Youth Sanjin Scholars, the 136 Medical Rejuvenation Project of Shanxi Province, the Multidisciplinary Team for Cognitive Impairment of Shanxi Science, Technology Innovation Training Team (201705D131027); Shanxi Provincial Science and technology achievements transformation and guidance project (201904d131020). |  |
| **Study** | **Mean age (*SD*)** | **Setting** | **Inclusion criteria** | **Exclusion criteria** | **AVATAR Protocol Info** | **Control Protocol Info** | **Diagnoses Distributions** | **Funding Information** |  |
| Percie du Sert et al. 2018^7^ | 42.9 (12.4) | Outpatient | Schizophrenia or  schizoaffective disorder (DSM-5); hearing persecutory voices;  no response to at least two antipsychotic trials. | Any change in medication within the past 2 months; substance use disorder (within the last 12 months), neurological disorder or unstable and serious physical illness; highly unstable state (e.g., currently in psychiatric intensive care unit); receiving CBT for psychosis within last 12 months | 7 weekly VR sessions AVATAR + TAU: immersion in dark virtual room from first person VR perspective  Session 1: avatar creation of most distressing entity of source of voice with peer patient,  Sessions 1-3: Exposure to hallucinatory experience; dialogue between patient and avatar sentences they provided; encouraged to improve emotional regulation and assertiveness;  Session 4: Self-esteem enabling participant to express themself and be aware of own qualities  Over course of treatment, avatar became less abusive and more supportive; empowerment in interaction participant developed more assertiveness  Final sessions: patients encouraged to apply what was learned in sessions | TAU + WL (no precise information) | 80% schizophrenia; 20% schizoaffective disorder | Philippe-Pinel Foundation, Fondation Jean-Louis Lévesque; Eli Lilly Canada Chair on Schizophrenia Research; the Applications de la Réalité Virtuelle en Psychiatrie Légale laboratory |  |
| **Study** | **Mean age (*SD*)** | **Setting** | **Inclusion criteria** | **Exclusion criteria** | **AVATAR Protocol Info** | **Control Protocol Info** | **Diagnoses Distributions** | **Funding Information** |  |
| Smith et al. 2025^8^ | Reported per group:   Challenge: 32.48 (11.6)   Control: 33.20 (12.2) | Outpatient | Age ≥ 18; diagnosis of a schizophrenia spectrum disorder (ICD-10); experienced auditory hallucinations for at least 3 months; scoring ≥ 3 on auditory hallucinations on the SANS; currently treated by a psychiatric outpatient facility in Denmark; able to give informed consent; unchanged antipsychotic medications within past four weeks; patient either not responded sufficiently to current antipsychotic treatment or no longer in antipsychotic treatment but had previously tried minimum of two antipsychotics without sufficient treatment response | Unable to identify a dominant voice; diagnosed with organic brain disease; substance abuse leading to non-attendance of treatment, or the person being intoxicated for treatment sessions; auditory hallucinations in language not spoken by the therapists; patient’s command of spoken Danish or English inadequate for engaging in therapy; person not able to tolerate assessment process; suffering from strongly impaired vision | 7 sessions of VR-based AVATAR in three phases; VR-dialogues lasted 2-20 of 60 minutes:  Phase 1: reclaiming power and control over the voice  Phase 2: self-worth  Phase 3: recovery and hope for the future.  Session 1: Creation of avatar for dominant voice;  Therapy progression: the avatar becomes less powerful and more compassionate  Post-sessions: Animated beach with accompanying wave sounds, designed for participants to use after VR-exposure or at the end of a session | TAU extended: Non-manualized supportive sessions under Danish OPUS Early  Intervention Services for First-Episode Psychosis or Flexible-Assertive Community Treatment Teams (minimum 7 sessions) + TAU: TAU extended | 90.4% schizophrenia; 1.5% Persistent delusional disorders; 2.2% schizoaffective disorder; 4.1% Other nonorganic psychotic disorders (F28 and F29) | Innovation Fund Denmark; Independent Research Fund Denmark; Innovation Fund North Denmark Research Fund North Denmark Region; M. L. Jørgensen and Gunnar Hansen Fund Funding Sources; Study leaders have received compensation from Heka VR for teaching AVATAR VRT to clinicians in other research projects |  |
| **Study** | **Mean age (*SD*)** | **Setting** | **Inclusion criteria** | **Exclusion criteria** | **AVATAR Protocol Info** | **Control Protocol Info** | **Diagnoses Distributions** | **Funding Information** |  |
| Stefaniak et al. 2019^9^ | 33.2 (7.3) | Mixed | Suffering chronically from auditory hallucinations; having undergone long-lasting psychiatric treatment | NI | 8 Sessions screen-based AVATAR + TAU; therapist in same room as avatar; no avatar creation  Session 1: Characterizing AVH; identification of exacerbating/relaxing situations; AVH content; present strategies  Session 2: Cognitive models; beliefs about origins of voices; relation between AVH and life  Session 3: Introducing avatar; recounting first AVH; explaining method; encouragement to reply with higher assertiveness  Session 4-6: Developing dialog between participant and avatar; creating conceptualization of AVH; early avatar content is negative; gradually become more neutral; modifying beliefs; meaning of AVH content; references of AVH content to relationships  Session 7: Modification of patient beliefs about voices and dysfunctional schemata. Increasing patients’ controllability over AVH; new strategies and meanings; consolidation of skills  Session 8: Summary of therapy | Waitlist (+TAU) maintenance of antipsychotics and their dosage | 96% schizophrenia; 4% Paranoid personality disorder | NI |  |

# **Table S7**. Additional Information Received from Study Authors

| **Study** | **Additional Information Received** |
| --- | --- |
| Leff et al. 2013 | PSYRATS-AH frequency and distress subscales; information on participant ages |
| Percie du Sert et al. 2018 | Between-group data before crossover; information about randomization |
| Smith et al. 2025 | Group-specific SEs |
| Stefaniak et al. 2019 | Between-group data before crossover and PSYRATS-AH frequency and distress subscales |

# **Table S8**. ROB-2 Ratings

| **Study** | **1.1**  **Was the allocation**  **sequence random?** | | | | **1.2 Was the allocation**  **sequence concealed until**  **participants were**  **enrolled and assigned to**  **interventions?** | | | | **1.3 Did baseline**  **differences between**  **intervention groups**  **suggest a problem with**  **the randomization**  **process?** | | | **1.0 Judgement** | |
| --- | --- | --- | --- | --- | --- | --- | --- | --- | --- | --- | --- | --- | --- |
| Craig et al. 2018 | Y | | | | Y | | | | N | | | Low | |
| Dellazizzo et al. 2021 | NI | | | | PY | | | | N | | | Low | |
| Garety et al. 2024 | Y | | | | Y | | | | N | | | Low | |
| Leff et al. 2013 | NI | | | | PY | | | | N | | | Low | |
| Liang et al. 2022 | Y | | | | NI | | | | N | | | Some concerns | |
| Percie du Sert et al. 2018 | N | | | | N | | | | Y | | | High | |
| Smith et al. 2025 | Y | | | | Y | | | | N | | | Low | |
| Stefaniak et al. 2019 | N | | | | PN | | | | PN | | | High | |
|  | | | | | | | | | | | | | |
| **Study** | **2.1 Were participants**  **aware of their assigned**  **intervention during the**  **trial?** | | **2.2 Were carers and**  **people delivering the**  **interventions aware of**  **participants assigned**  **intervention during the**  **trial?** | | **2.3 Were there**  **deviations from the**  **intended intervention**  **that arose because of the**  **trial context?** | | **2.4 Were**  **these deviations likely to**  **have affected the**  **outcome?** | | **2.5 Were these deviations**  **from intended**  **intervention balanced**  **between groups?** | **2.6 Was an appropriate**  **analysis used to estimate**  **the effect of assignment**  **to intervention?** | | **2.7 Was there potential for a**  **substantial impact (on**  **the result) of the failure**  **to analyse participants in**  **the group to which they**  **were randomized?** | **2.0 Judgement** |
| Craig et al. 2018 | Y | | Y | | n | | NA | | NA | y | | NA | low |
| Dellazizzo et al. 2021 | Y | | Y | | n | | NA | | NA | y | | NA | low |
| Garety et al. 2024 | Y | | Y | | n | | NA | | NA | y | | NA | low |
| Leff et al. 2013 | Y | | Y | | n | | NA | | NA | y | | NA | low |
| Liang et al. 2022 | Y | | Y | | n | | NA | | NA | y | | NA | low |
| Percie du Sert et al. 2018 | Y | | Y | | n | | NA | | NA | y | | NA | low |
| Smith et al. 2025 | Y | | Y | | n | | NA | | NA | y | | NA | low |
| Stefaniak et al. 2019 | Y | | Y | | n | | NA | | NA | y | | NA | low |
|  |  | |  | |  | |  | |  |  | |  |  |
| **Study** | **3.1 Were data for this**  **outcome available for all,**  **or nearly all, participants**  **randomized?** | | | | **3.2 Is**  **there evidence that the**  **result was not biased by**  **missing outcome data?** | | | **3.3 Could**  **missingness in the**  **outcome depend on its**  **true value?** | | **3.4 Is it**  **likely that missingness in**  **the outcome depended on**  **its true value?** | | | **3.0 judgement** |
| Craig et al. 2018 | N | | | | N | | | PY | | PY | | | High |
| Dellazizzo et al. 2021 | N | | | | PN | | | PY | | PY | | | High |
| Garety et al. 2024 | N | | | | Y | | | NA | | NA | | | Low |
| Leff et al. 2013 | N | | | | N | | | Y | | PY | | | High |
| Liang et al. 2022 | N | | | | N | | | PY | | PY | | | High |
| Percie Du Sert et al. 2018 | N | | | | N | | | Y | | Y | | | High |
| Smith et al. 2025 | N | | | | PY | | | NA | | NA | | | Low |
| Stefaniak et al. 2019 | N | | | | N | | | Y | | Y | | | High |
|  | | | | | | | | | | | | | |
| **Study** | | **4.1 Was the method of**  **measuring the outcome**  **inappropriate?** | | **4.2 Could measurement**  **or ascertainment of the**  **outcome have differed**  **between intervention**  **groups?** | | **4.3 Were outcome**  **assessors aware of the**  **intervention received by**  **study participants?** | | | **4.4 Could assessment of the**  **outcome have been**  **influenced by knowledge**  **of intervention received?** | | **4.5 Is it**  **likely that assessment of**  **the outcome was**  **influenced by knowledge**  **of intervention received?** | | **4.0 result** |
| Craig et al. 2018 | | n | | n | | N | | | NA | | NA | | Low |
| Dellazizzo et al. 2021 | | n | | n | | Y | | | PY | | PY | | High |
| Garety et al. 2024 | | n | | n | | N | | | NA | | NA | | Low |
| Leff et al. 2013 | | n | | n | | N | | | NA | | NA | | Low |
| Liang et al. 2022 | | n | | n | | N | | | NA | | NA | | Low |
| Percie du Sert et al. 2018 | | n | | n | | Y | | | Y | | PY | | High |
| Smith et al. 2025 | | n | | n | | N | | | NA | | NA | | Low |
| Stefaniak et al. 2019 | | n | | n | | Y | | | Y | | PY | | High |
|  | | | | | | | | | | | | | |
| **Study** | **5.1 Were the data that**  **produced this result**  **analyzed in accordance with a pre-specified analysis plan that was finalized before unblinded outcome data were available for analysis?** | | | | **5.2 Selected on the basis of results from multiple eligible**  **outcome measurements**  **(e.g. scales, definitions,**  **time points) within the**  **outcome domain?** | | | **5.3 Selected on the basis of multiple eligible**  **analyses of the data?** | | **5.0 Algorithm result** | | | **Overall bias** |
| Craig et al. 2018 | Y | | | | N | | | N | | Low | | | High |
| Dellazizzo et al. 2021 | NI | | | | PN | | | PN | | Some concerns | | | High |
| Garety et al. 2024 | Y | | | | N | | | N | | Low | | | Low |
| Leff et al. 2013 | NI | | | | N | | | N | | Some concerns | | | High |
| Liang et al. 2022 | NI | | | | PN | | | PN | | Some concerns | | | High |
| Percie u Sert et al. 2018 | NI | | | | N | | | N | | Some concerns | | | High |
| Smith et al. 2025 | Y | | | | N | | | N | | Low | | | Low |
| Stefaniak et al. 2019 | NI | | | | N | | | N | | Some concerns | | | High |

*Note.* n = no; NI = no information; pn = probably no; py = probably yes; y = yes.

#

# **Table S9**. Grading of Recommendations Assessment, Development and Evaluation (GRADE)

| **Outcome** | **Number of Studies and Participants** | **Comments** | **Hedges *g* and 95% CI** | **Certainty Rating** |
| --- | --- | --- | --- | --- |
| **AVH severity at post-treatment** | 9 randomized controlled trials  EG 462 vs. CG 352 participants | - Often high bias (6/9 comparisons) but smaller trials showed higher risk of bias; large trials with majority of participants showed low risks of bias; sensitivity analyses of studies with low risk of bias show similar effects 🡪 not downgraded - Egger's test significant 🡪 rated down one grade | −0.40 [−0.54, −0.25] | Moderate |
| **AVH severity at 12-week follow-up** | 6 randomized controlled trials  EG 418 vs. CG 319 participants | - Often high bias (3/6 comparisons) Smaller trials showed higher risk of bias; large trials with majority of participants showed low risks of bias; sensitivity analyses of studies with low risk of bias show similar effects - Number of participants smaller than OIS of 788 (=737) 🡪 rated down one grade - Crosses over into range of non-minimally important difference (SMD = -0.2) 🡪 rated down one grade | −0.25 [−0.40, −0.10] | Low |
| **AVH severity at 24-week follow-up** | 1 randomized controlled trial  EG 13 vs. CG 20 participants | - Single study with high bias in multiple domains 🡪 rated down two grades - Number of participants smaller than OIS of 788 (33) 🡪 rated down two grades - Crosses into range of possible harm (SMD = 0.2) 🡪 rated down two grades | –0.30 [–1.00, 0.40] | Very low |
| **AVH severity at 52-week follow-up** | 1 randomized controlled trial  EG 13 vs. CG 17 participants | - Single study with high bias in multiple domains 🡪 rated down two grades - Number of participants smaller than OIS of 788 (30) 🡪 rated down two grades - Crosses into range of possible harm (SMD = 0.2) 🡪 rated down two grades | –0.26 [–0.99, 0.46] | Very low |
| **AVH frequency at post-treatment** | 9 randomized controlled trials  EG 462 vs. CG 352 participants | - Often high bias (6/9 comparisons) but smaller trials showed higher risk of bias; large trials with majority of participants showed low risks of bias; sensitivity analyses of studies with low risk of bias show similar effects 🡪 not downgraded | −0.38 [−0.52, −0.24] | High |
| **AVH frequency at 12-week follow-up** | 6 randomized controlled trials  EG 421 vs. CG 320 participants | - Often high bias (3/6 comparisons) but smaller trials showed higher risk of bias; large trials with majority of participants showed low risks of bias; sensitivity analyses of studies with low risk of bias show similar effects 🡪 not downgraded - Number of participants smaller than OIS of 788 (=741) 🡪 rated down one grade - Crosses over into range of non-minimally important difference (SMD = -0.2) 🡪 rated down one grade | −0.34 [−0.49, −0.19] | Low |
| **AVH frequency at 24-week follow-up** | 1 randomized controlled trial  EG 13 vs. CG 20 participants | - Single study with high bias in multiple domains 🡪 rated down two grades - Number of participants smaller than OIS of 788 (33) 🡪 rated down two grades - Crosses into range of possible harm (SMD = 0.2) 🡪 rated down two grades | –0.33 [–1.03, 0.37] | Very low |
| **AVH frequency at 52-week follow-up** | 1 randomized controlled trial  EG 13 vs. CG 17 participants | - Single study with high bias in multiple domains 🡪 rated down two grades - Number of participants smaller than OIS of 788 (30) 🡪 rated down two grades - Crosses into range of possible harm (SMD = 0.2) 🡪 rated down two grades | –0.40 [–1.12, 0.33] | Very low |
| **AVH distress at post-treatment** | 9 randomized controlled trials  EG 462 vs. CG 352 participants | - Often high bias (6/9 comparisons) but smaller trials showed higher risk of bias; large trials with majority of participants showed low risks of bias; sensitivity analyses of studies with low risk of bias show similar effects 🡪 not downgraded - Egger's test significant 🡪 rated down one grade - Crosses over into range of non-minimally important difference (SMD = -0.2) 🡪 rated down one grade | −0.32 [−0.46, –0.18] | Low |
| **AVH distress at 12-week follow-up** | 6 randomized controlled trials  EG 419 vs. CG 320 participants | - Often high bias (3/6 comparisons) Smaller trials showed higher risk of bias; large trials with majority of participants showed low risks of bias; sensitivity analyses of studies with low risk of bias showed non-significant effects 🡪 rated down one grade - Number of participants smaller than OIS of 788 (739) 🡪 rated down one grade - Crosses over into range of non-minimally important difference (SMD = -0.2) 🡪 rated down one grade | −0.20 [−0.35, −0.06] | Very low |
| **AVH distress at 24-week follow-up** | 1 randomized controlled trial  EG 13 vs. CG 20 participants | - Single study with high bias in multiple domains 🡪 rated down two grades - Number of participants smaller than OIS of 788 (33) 🡪 rated down two grades - Crosses into range of possible harm (SMD = 0.2) 🡪 rated down two grades | –0.21 [–0.91, 0.49] | Very low |
| **AVH distress at 52-week follow-up** | 1 randomized controlled trial  EG 13 vs. CG 17 participants | - Single study with high bias in multiple domains 🡪 rated down two grades - Number of participants smaller than OIS of 788 (30) 🡪 rated down two grades - Crosses into range of possible harm (SMD = 0.2) 🡪 rated down two grades | –0.18 [–0.90, 0.54] | Very low |

*Note.* Rated using GRADEPro (McMaster University and Evidence Prime, 2025). The optimal information size (OIS) required for the assessment of precision in GRADE was calculated to be a total sample size of 788 participants using g*power (power = 0.8, α = .05, Cohen’s d = 0.2; Faul et al., 2007; Zeng et al., 2022).

# **Table S10.** Included Measurements by Outcome and Study

| **Study** | **Outcome of Interest** |
| --- | --- |
| **Craig et al. 2018** | AVH severity: PSYRATS-AH^10^  AVH frequency: PSYRATS-AH frequency subscale^10^  AVH distress: PSYRATS-AH distress subscale^10^  Positive symptoms: SAPS^11^  Negative symptoms: SANS^12^  Depressive symptoms: DASS-21^13^  Anxiety symptoms: DASS-21^13^  Quality of life: MANSA^14^ |
| **Dellazizzo et al. 2021** | AVH severity: PSYRATS-AH^10^  AVH frequency: PSYRATS-AH frequency subscale^10^  AVH distress: PSYRATS-AH distress subscale^10^  Depressive symptoms: BDI-II^15^  Positive symptoms: PANSS positive subscale^15,16^  Negative symptoms: PANSS negative subscale^16^  Total psychotic symptoms: PANSS^16^  Quality of life: Q-LES-Q-SF^17^ |
| **Garety et al. 2024** | AVH distress: PSYRATS-AH distress subscale^10^  AVH frequency: PSYRATS-AH frequency subscale^10^  AVH severity: PSYRATS-AH^10^  Positive symptoms: PSYRATS-AH / PSYRATS-D aggregated score^10^  Depressive symptoms: DASS-21^13^  Anxiety symptoms: DASS-21^13^ |
| **Leff et al. 2013** | AVH severity: PSYRATS-AH^10^  AVH distress: PSYRATS-AH distress subscale^10^  AVH frequency: PSYRATS-AH frequency subscale^10^  Depressive symptoms: CDS^18^ |
| **Liang et al. 2022** | AVH distress: PSYRATS-AH distress subscale^10^  AVH frequency: PSYRATS-AH frequency subscale^10^  AVH severity: PSYRATS-AH^10^  Positive symptoms: PANSS^16^  Negative symptoms: PANSS ^16^  Total psychotic symptoms: PANSS^16^  Quality of life: Q-LES-Q-SF^17^  Depressive symptoms: HAMD^19^  Anxiety symptoms: HAMA^20^ |
| **Percie du Sert et al. 2018** | AVH severity: PSYRATS-AH^10^  AVH frequency: PSYRATS-AH frequency subscale^10^  AVH distress: PSYRATS-AH distress subscale^10^  Positive symptoms: PANSS^16^  Negative symptoms: PANSS^16^  Total psychotic symptoms: PANSS^16^  Depressive symptoms: BDI-II^15^  Quality of life: Q-LES-Q-SF^17^ |
| **Smith et al. 2025** | AVH severity: PSYRATS-AH^21^  AVH frequency: PSYRATS-AH frequency subscale^10^  AVH distress: PSYRATS-AH distress subscale^10^  Positive symptoms: SAPS^11^  Negative symptoms: BNSS^22^  Depressive symptoms: CDS^18^  Social functioning: SFS^21^ |
| **Stefaniak et al. 2019** | AVH severity: PSYRATS-AH^10^  AVH frequency: PSYRATS-AH frequency subscale^10^  AVH distress: PSYRATS-AH distress subscale^10^  Positive symptoms: PSYRATS total score^10^ |

# **Table S11**. Statistics for Publication Bias Tests

| **Outcome** | **Timeframe / analysis type** | **Number of studies** | **Egger’s regression tests**^23^ |
| --- | --- | --- | --- |
| **AVH Severity** | T1 | 9 | *z =* −2.60*, p =* 0.009** |
|  | T2 | 6 | *z =* −0.63*, p =* 0.53 |
| **AVH Frequency** | T1 | 9 | *z* = −1.00, *p* = 0.32 |
|  | T2 | 6 | *z* = −0.10, *p* = 0.92 |
| **AVH Distress** | T1 | 9 | *z* = −2.29, *p* = .02* |
|  | T2 | 6 | *z* = 0.10, *p* = 0.92 |
| **Total psychotic symptoms** | T1 | 3 | *z* = −0.97, *p* = 0.33 |
|  | T2 | 2 | NA |
| **Positive symptoms** | T1 | 8 | *z* = −1.12, *p* = 0.26 |
|  | T2 | 6 | *z* = 0.74, *p* = 0.46 |
| **Negative symptoms** | T1 | 5 | *z* = −0.34, *p* = 0.73 |
|  | T2 | 4 | *z* = −0.16, *p* = 0.87 |
| **Depressive symptoms** | T1 | 8 | *z* = −0.50, *p* = 0.62 |
|  | T2 | 6 | *z* = 0.58, *p* = 0.56 |
| **Anxiety symptoms** | T1 | 4 | *z* = −0.47, *p* = 0.64 |
|  | T2 | 4 | *z* = 0.13, *p* = 0.90 |
| **Quality of Life** | T1 | 4 | *z* = 1.67, *p* = 0.09 |
|  | T2 | 3 | *z* = 0.34, *p* = 0.73 |
| **Treatment dropout** | Proportion in intervention groups | 9 | NA |
|  | Proportion in Control groups | 5 | NA |
|  | Risk ratio intervention vs. control groups |  | *z* = 0.18, *p* = 0.86 |
| **Study dropout** | Proportion in intervention groups | 9 | NA |
|  | Proportion in control groups |  | NA |
|  | Risk ratio intervention vs. control groups |  | *z* = 0.47, *p* = 0.64 |

*Note.* NA = Not Applicable; T1 = post-treatment; T2 = follow-up*.* * p < 0.05, ** p ≤ 0.01, *** p

^1^Not performable for meta-regression, generalized mixed models, or k < 3

# **Figure S1**. PRISMA Screening Flow Diagram


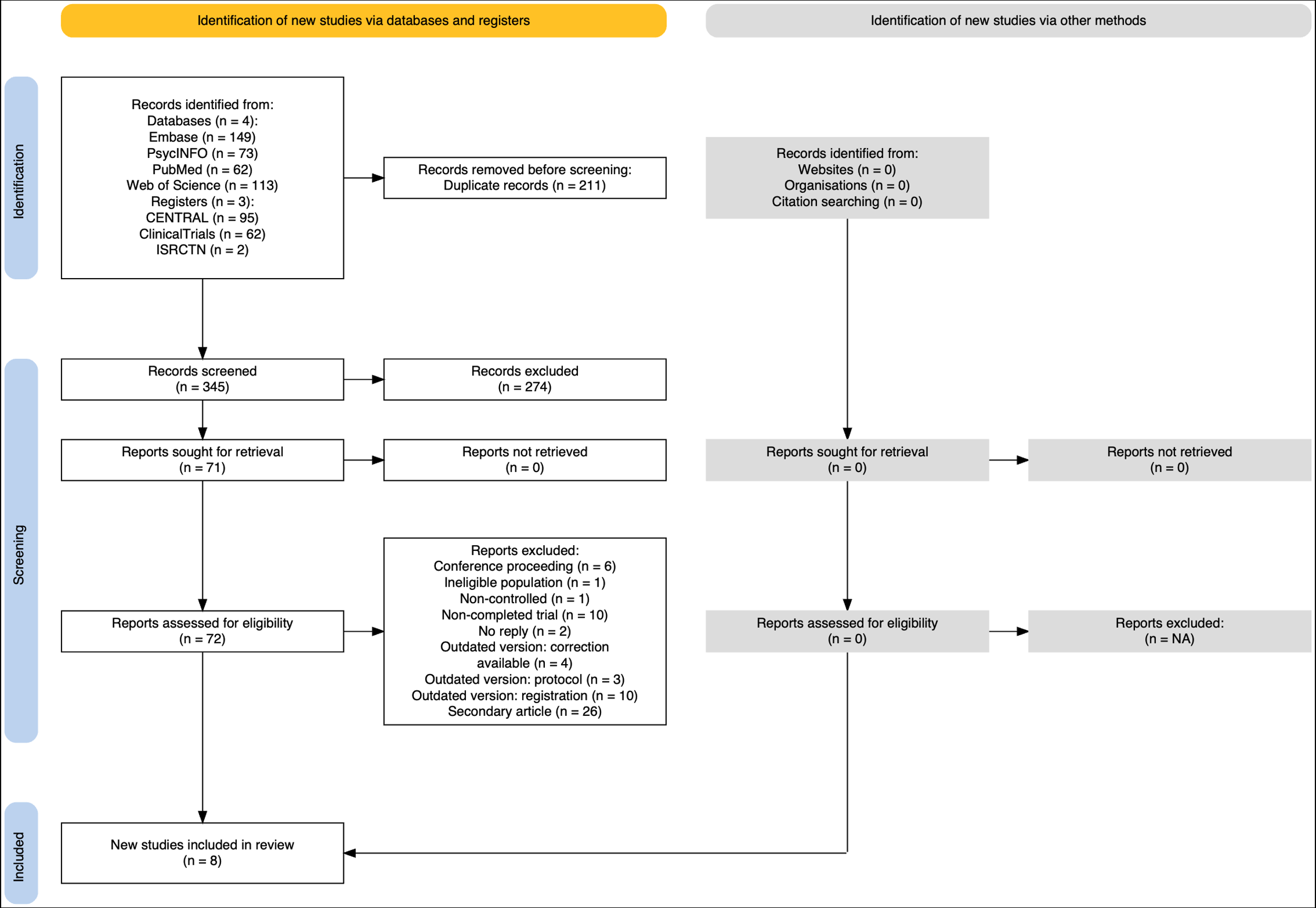


*Note.* Created with PRISMA2020 Shiny App.^24^

# **Figure S2**. Forest Plots of AVH Frequency at Post-Treatment and Short-Term Follow-up


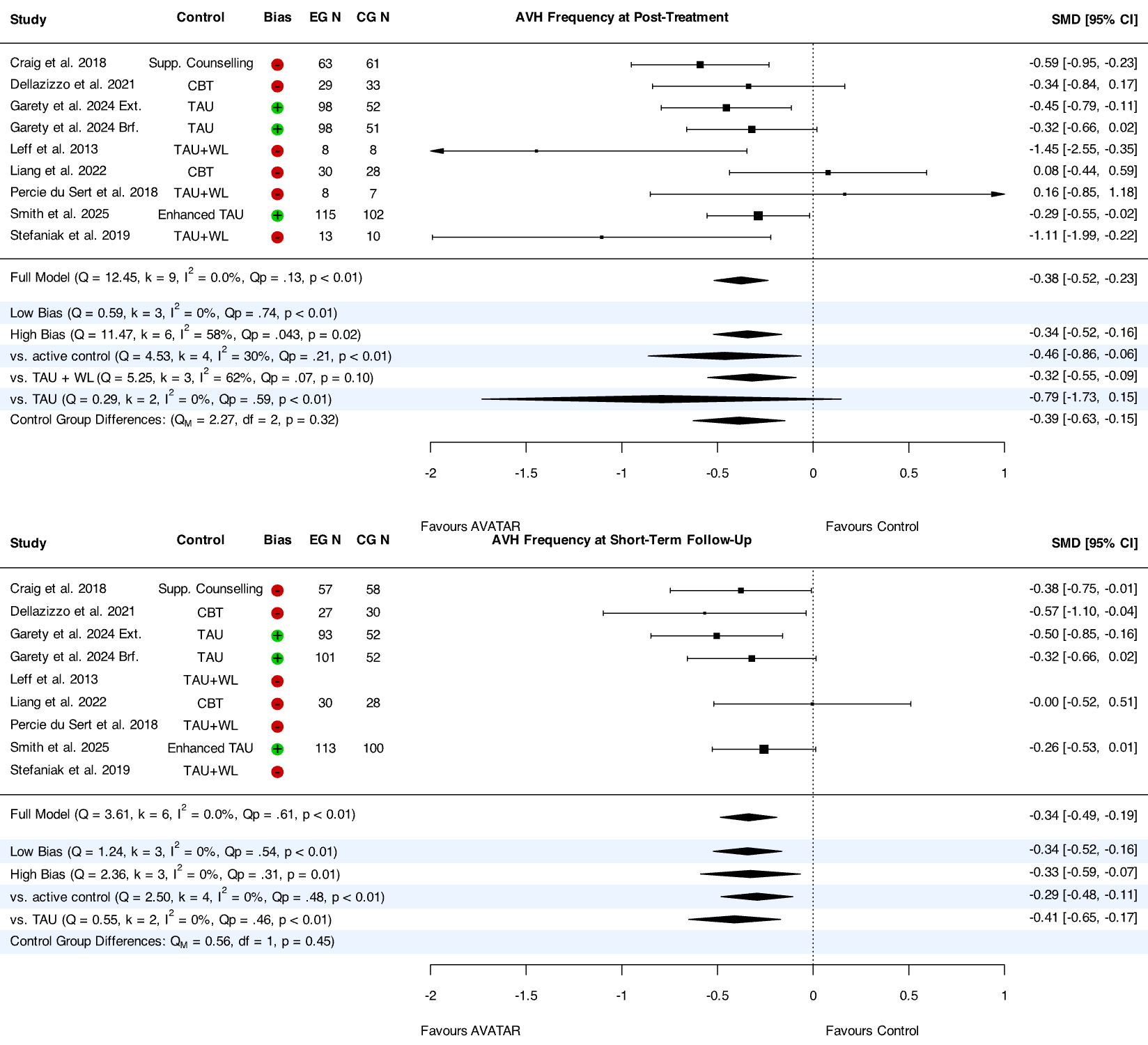


*Note.* Negative SMDs portray smaller means in AVATAR compared to control groups. ⊕ = Low bias; ⊖ = high bias; CBT = cognitive behavioral therapy; CG = control group; EG = experimental group; I^2^ = Higgins’ heterogeneity statistic; k = number of studies; N = sample size; Q = Cochran’s Q statistic; Qp = Cochran’s Q *p-*value; SMD = standardized mean difference in Hedges’ g; supp. = supportive. TAU = treatment as usual.

# **Figure S3**. Forest Plots of AVH Distress at Post-Treatment and Short-Term Follow-up


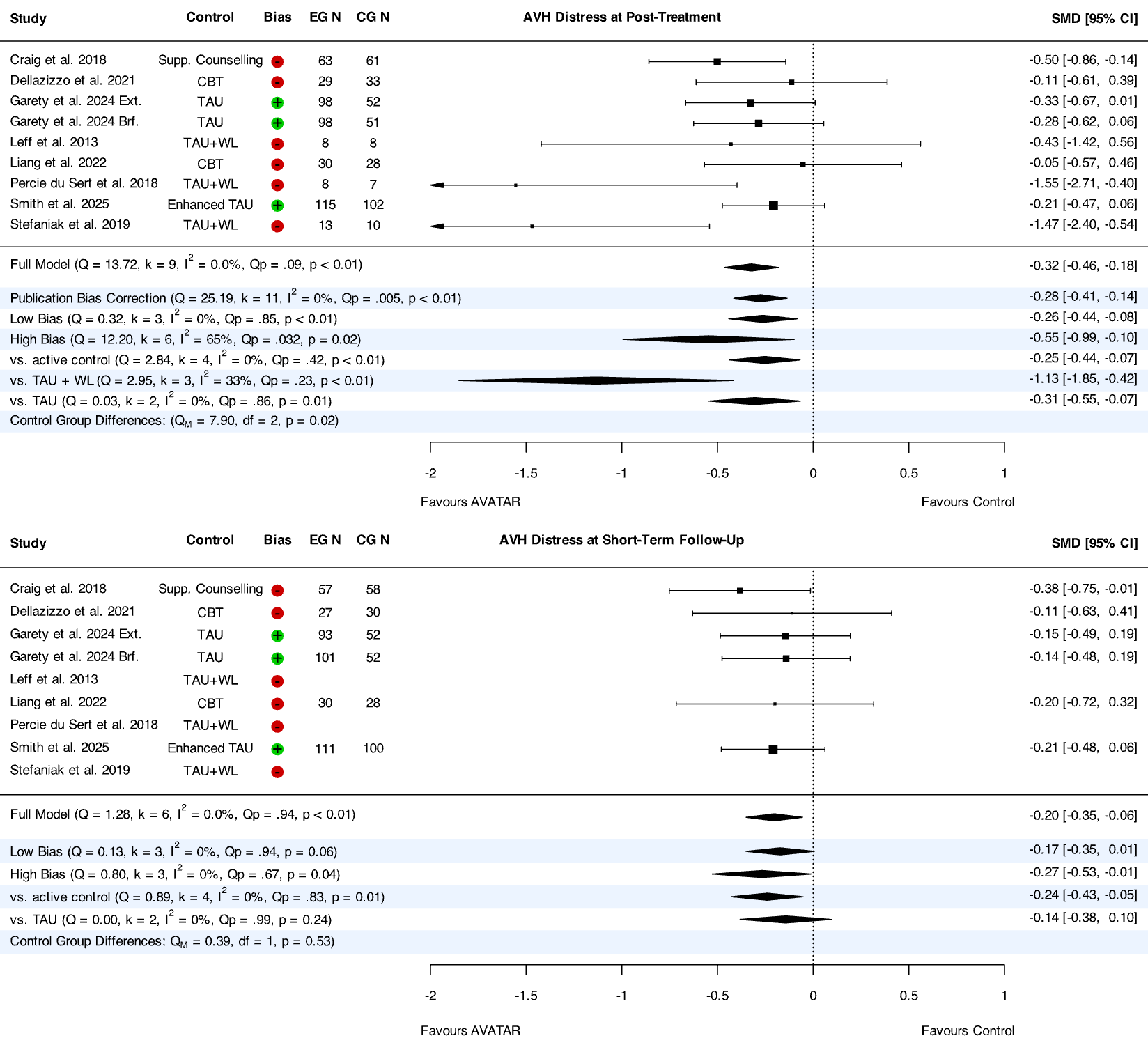


*Note.* Negative SMDs portray smaller means in AVATAR compared to control groups. ⊕ = Low bias; ⊖ = high bias; CBT = cognitive behavioral therapy; CG = control group; EG = experimental group; I^2^ = Higgins’ heterogeneity statistic; k = number of studies; N = sample size; Q = Cochran’s Q statistic; Qp = Cochran’s Q *p-*value; SMD = standardized mean difference in Hedges’ g; supp. = supportive. TAU = treatment as usual.

# **Figure S4**. Forest Plot of Psychotic Symptoms at Post-Treatment and Follow-up


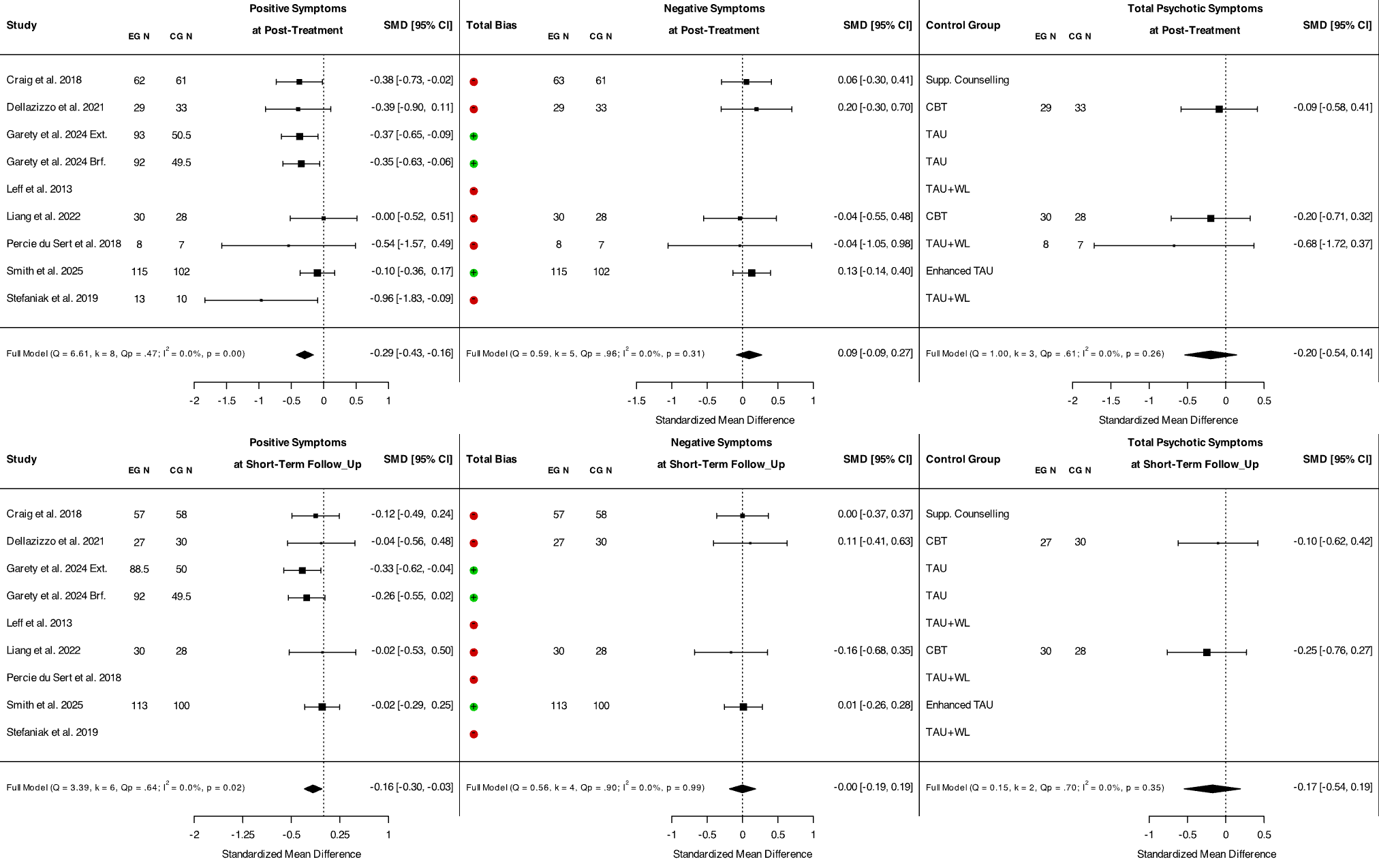


*Note.* Negative SMDs portray smaller means in AVATAR compared to control groups. ⊕ = Low bias; ⊖ = high bias; CBT = cognitive behavioral therapy; CG = control group; EG = experimental group; I^2^ = Higgins’ heterogeneity statistic; k = number of studies; N = sample size; Q = Cochran’s Q statistic; Qp = Cochran’s Q *p-*value; SMD = standardized mean difference in Hedges’ g; supp. = supportive. TAU = treatment as usual.

# **Figure S5**. Forest Plot of Anxiety Symptoms, Depressive Symptoms, and Quality of Life at Post-Treatment and Follow-up


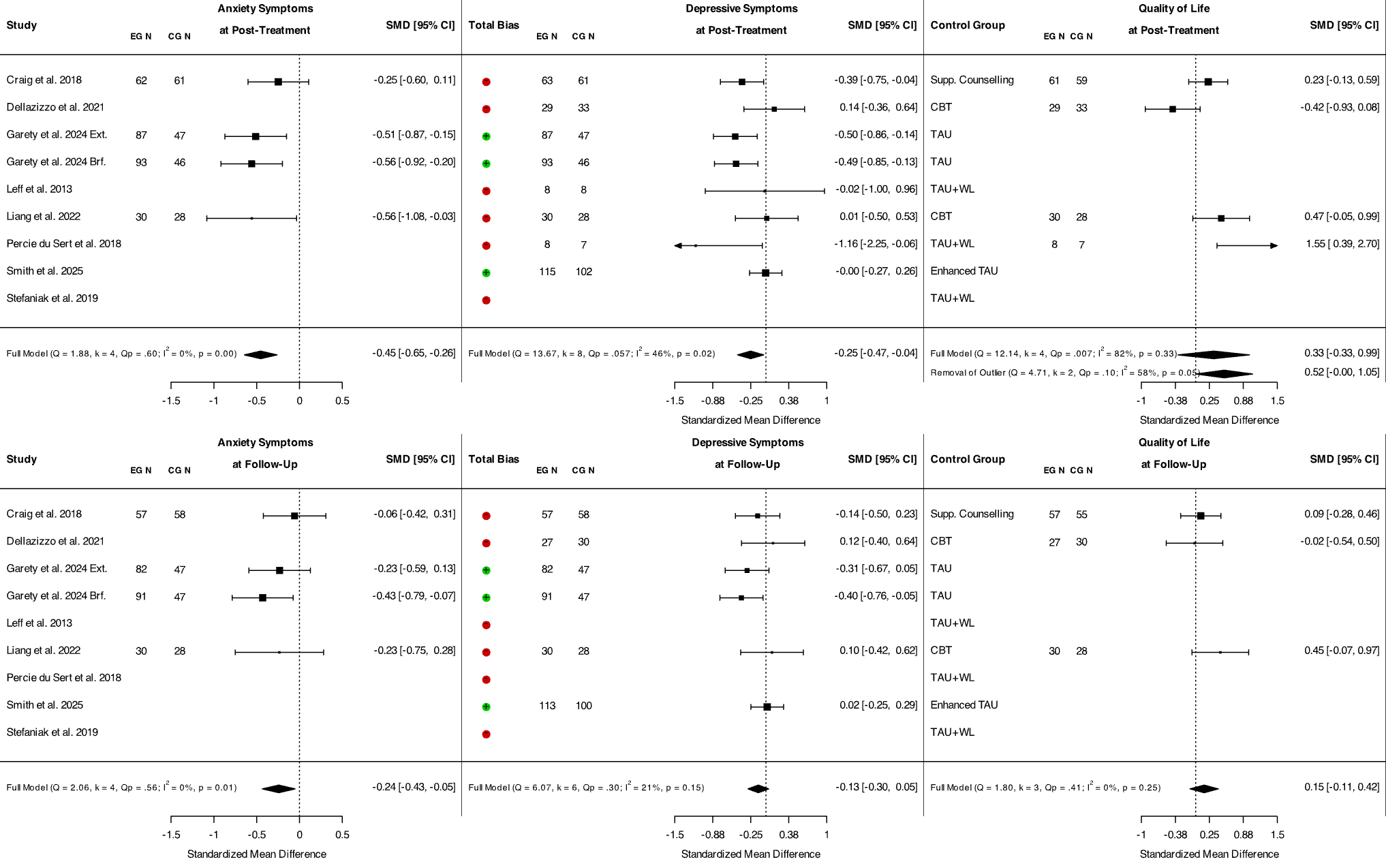


*Note.* Negative SMDs portray smaller means in AVATAR compared to control groups. ⊕ = Low bias; ⊖ = high bias; CBT = cognitive behavioral therapy; CG = control group; EG = experimental group; I^2^ = Higgins’ heterogeneity statistic; k = number of studies; N = sample size; Q = Cochran’s Q statistic; Qp = Cochran’s Q *p-*value; SMD = standardized mean difference in Hedges’ g; supp. = supportive. TAU = treatment as usual.

# **Figure S6**. Forest Plots of Treatment- and Study Dropout Proportion and Risk Ratio


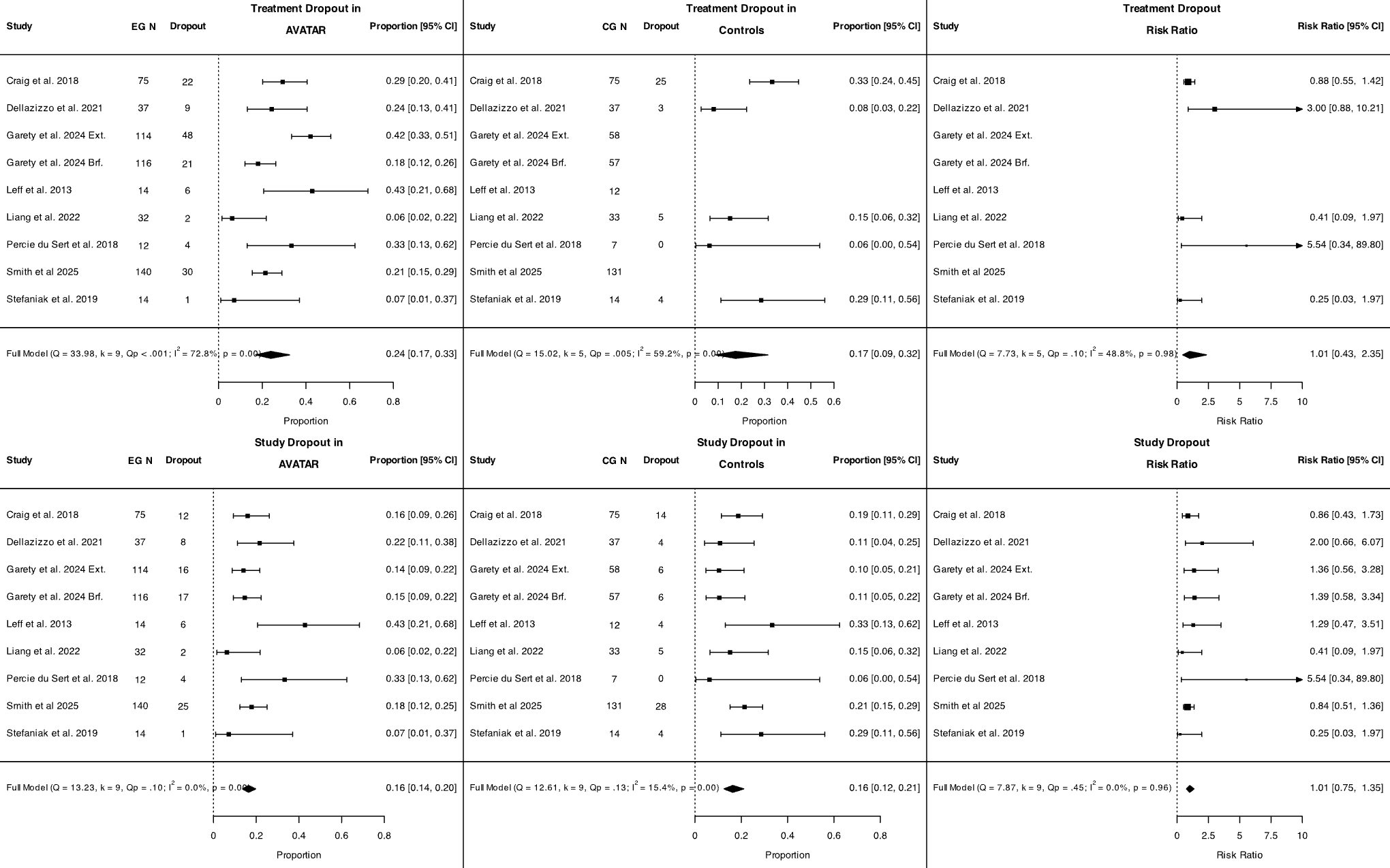


*Note*. A risk ratio larger than 1 described an increased risk of dropout in AVATAR compared to control groups. ⊕ = Low bias; ⊖ = high bias; CBT = cognitive behavioral therapy; CG = control group; EG = experimental group; I^2^ = Higgins’ heterogeneity statistic; k = number of studies; N = sample size; Q = Cochran’s Q statistic; Qp = Cochran’s Q *p-*value; SMD = standardized mean difference in Hedges’ g; supp. = supportive. TAU = treatment as usual.

# **Figure S7**. Funnel Plots for Voice-Related Outcomes at Post-Treatment and Follow-Up


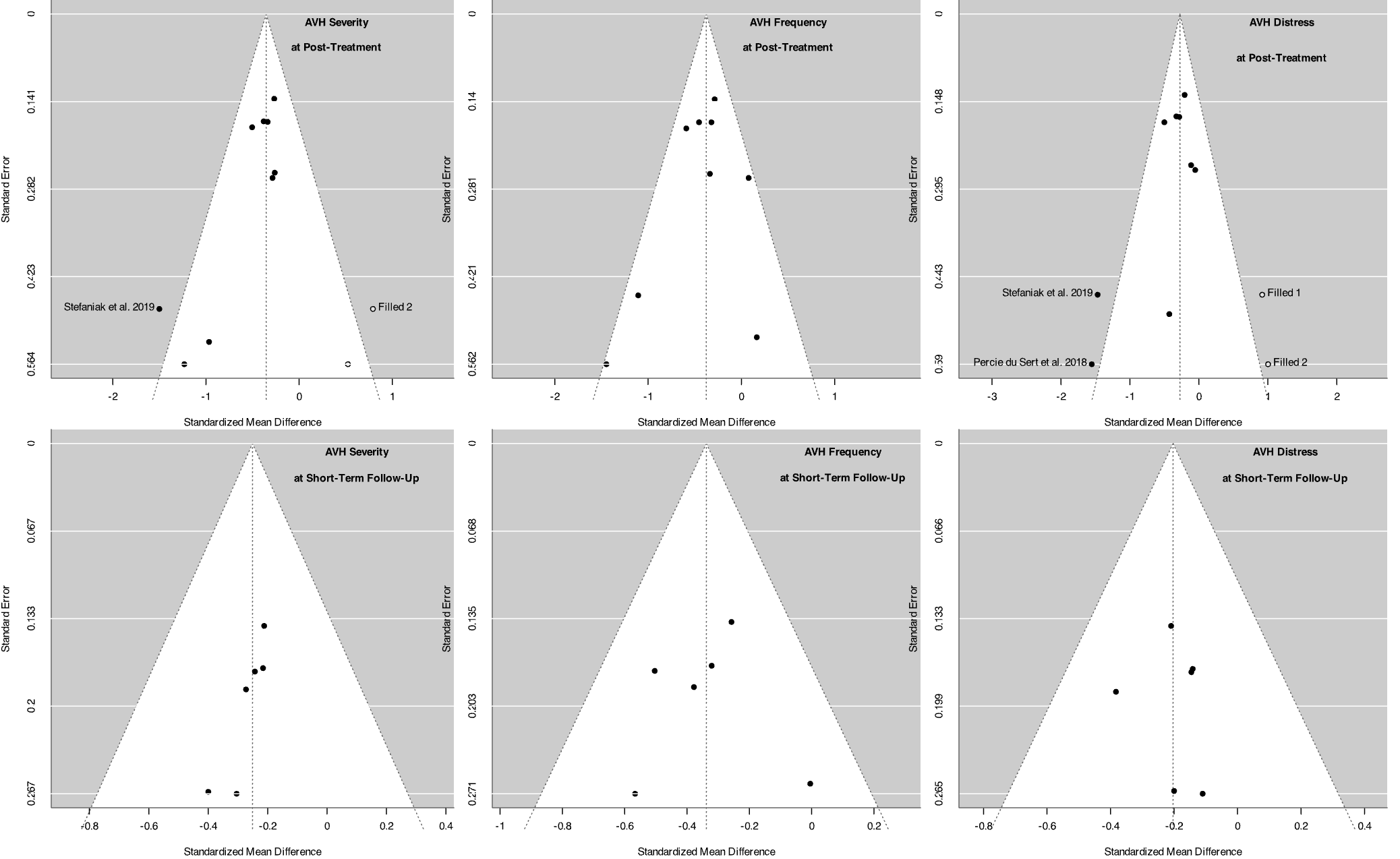


*Note.* Without publication bias, the effect sizes of the studies would be distributed symmetrically, with larger studies appearing toward the top and smaller studies toward the bottom of the graph. Grey dots show trim-and-fill estimated missing studies and labelled studies appear outside of the 95% CI of the standard error.

#

# **Figure S8**. Funnel Plots for Psychotic Symptoms at Post-Treatment and Follow-Up


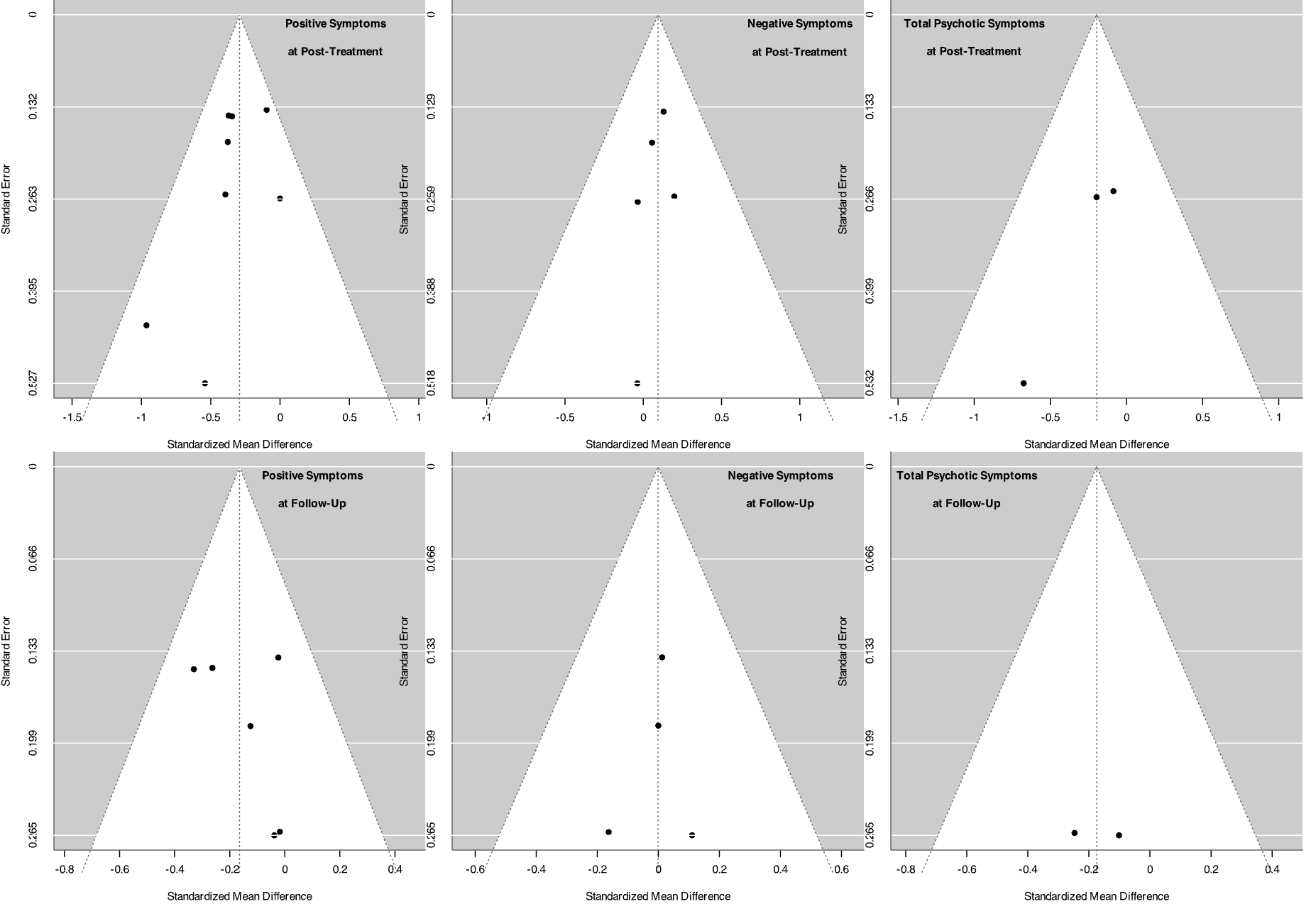


*Note.* Without publication bias, the effect sizes of the studies would be distributed symmetrically, with larger studies appearing toward the top and smaller studies toward the bottom of the graph. Labelled studies appear outside of the 95% CI of the standard error.

# **Figure S9**. Funnel Plots for Anxiety Symptoms, Depressive Symptoms, and Quality of Life at Post-Treatment and Follow-Up


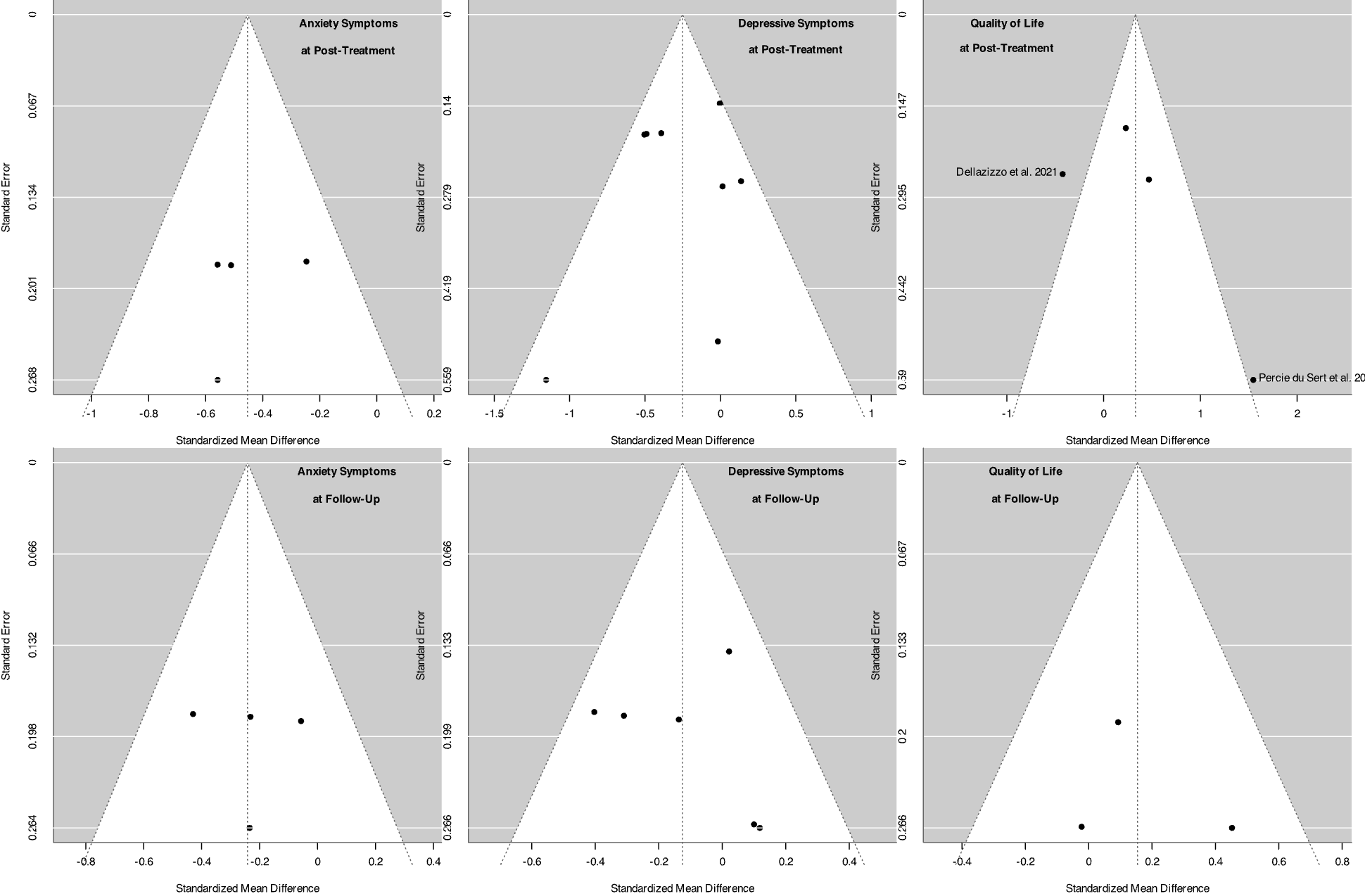


*Note.* Without publication bias, the effect sizes of the studies would be distributed symmetrically, with larger studies appearing toward the top and smaller studies toward the bottom of the graph. Labelled studies appear outside of the 95% CI of the standard error.

# **Figure S10**. Funnel Plots for Treatment- and Study Dropout Proportion and Risk Ratio


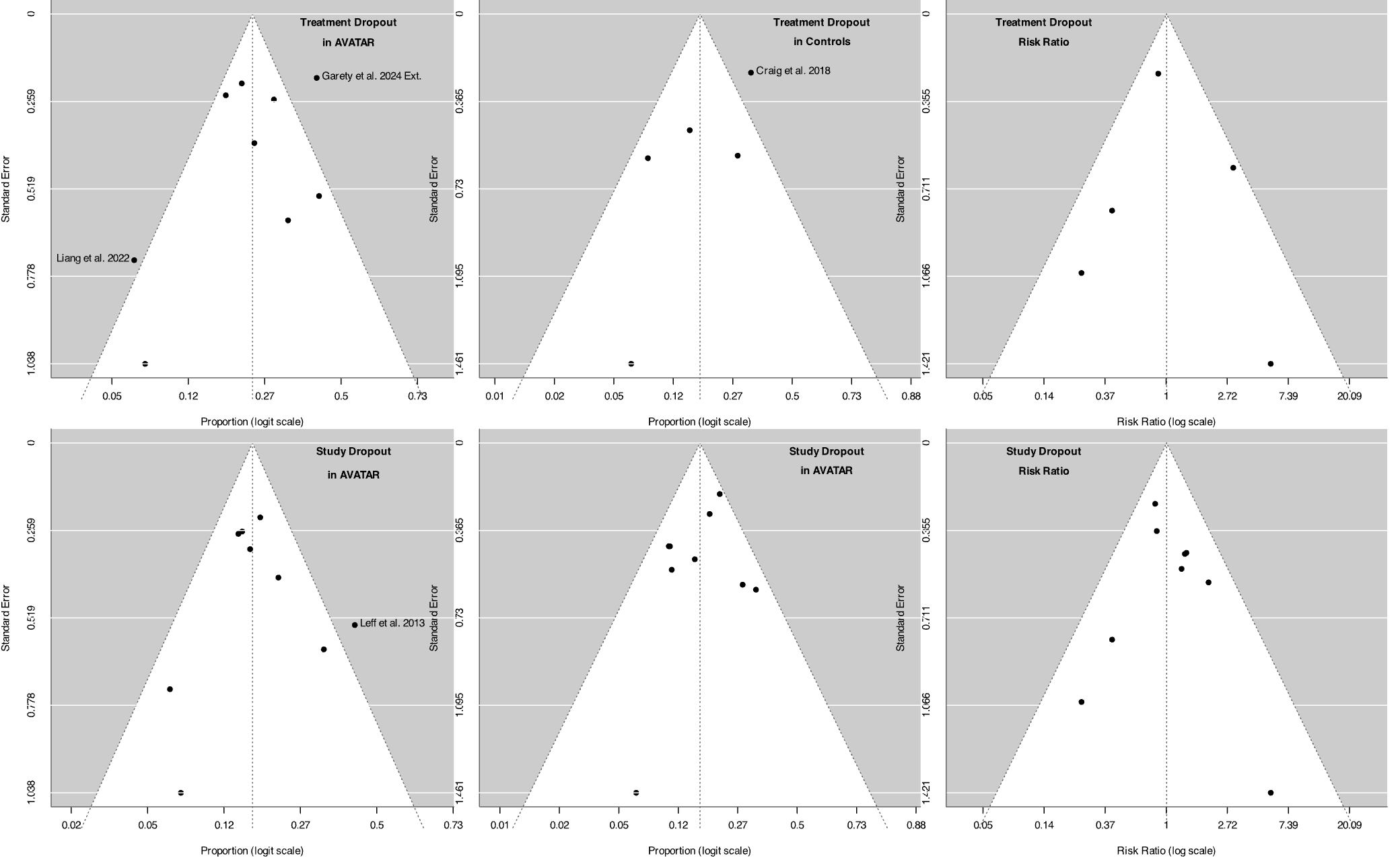


*Note.* Without publication bias, the effect sizes of the studies would be distributed symmetrically, with larger studies appearing toward the top and smaller studies toward the bottom of the graph. Labelled studies appear outside of the 95% CI of the standard error.

# **References**

1 Craig TKJ, Rus-Calafell M, Ward T, Leff JP, Huckvale M, Howarth E, *et al.* AVATAR therapy for auditory verbal hallucinations in people with psychosis: A single-blind, randomised controlled trial. *Lancet Psychiatry* 2018; **5**: 31–40.

2 Lewis S, Tarrier N, Haddock G, Bentall R, Kinderman P, Kingdon D, *et al.* Randomised controlled trial of cognitive-behavioural therapy in early schizophrenia: Acute-phase outcomes. *Br J Psychiatry* 2002; **181**: s91–7.

3 Dellazizzo L, Potvin S, Phraxayavong K, Dumais A. One-year randomized trial comparing virtual reality-assisted therapy to cognitive–behavioral therapy for patients with treatment-resistant schizophrenia. *NPJ Schizophr* 2021; **7**. doi:10.1038/s41537-021-00139-2.

4 Garety PA, Edwards CJ, Jafari H. Digital AVATAR therapy for distressing voices in psychosis: the phase 2/3 AVATAR2 trial. *Nat Med* 2024; **30**: 3658–68.

5 Leff J, Williams G, Huckvale MA, Arbuthnot M, Leff AP. Computer-assisted therapy for medication-resistant auditory hallucinations: proof-of-concept study. *Br J Psychiatry* 2013; **202**: 428–33.

6 Liang N, Li X, Guo X. Visual P300 as a neurophysiological correlate of symptomatic improvement by a virtual reality-based computer AT system in patients with auditory verbal hallucinations: A Pilot study. *J Psychiatr Res* 2022; **151**: 261–71.

7 Percie Du Sert O, Potvin S, Lipp O, Dellazizzo L, Laurelli M, Breton R, *et al.* Virtual reality therapy for refractory auditory verbal hallucinations in schizophrenia: A pilot clinical trial. *Schizophr Res* 2018; **197**: 176–81.

8 Smith LC, Vernal DL, Mariegaard LS, Christensen AG, Jansen JE, Schytte G, *et al.* Smith, L. C., Vernal, D. L., Mariegaard, L. S., Christensen, A. G., Jansen, J. E., Schytte, G., Stokbro, L. A., Albert, N., Christensen, M. J., Thomas, N., Hjorthøj, C., Nordentoft, M., & Glenthøj, L. B. Immersive virtual reality-assisted therapy targeting persistent auditory verbal hallucinations in patients diagnosed with schizophrenia spectrum disorders in Denmark: The Challenge assessor masked, randomized clinical trial. 2025 (in press).

9 Stefaniak I, Sorokosz K, Janicki A, Wciórka J. Therapy based on avatar-therapist synergy for patients with chronic auditory hallucinations: A pilot study. *Schizophr Res* 2019; **211**: 115–7.

10 Haddock G, McCARRON J, Tarrier N, Faragher EB. Scales to measure dimensions of hallucinations and delusions: the psychotic symptom rating scales (PSYRATS). *Psychol Med* 1999; **29**: 879–89.

11 Andreason NC. *The Scale for the Assessment of Positive Symptoms (SAPS).* University of Iowa, 1984.

12 Andreason NC. *The Scale for the Assessment of Negative Symptoms (SANS).* University of Iowa, 1984.

13 Lovibond PF, Lovibond SH. The structure of negative emotional states: comparison of the Depression Anxiety Stress Scales (DASS) with the Beck Depression and Anxiety Inventories. *Behav Res Ther* 1995; **33**: 335–43.

14 Priebe S, Huxley P, Knight S, Evans S. Application and Results of the Manchester Short Assessment of Quality of Life (Mansa). *Int J Soc Psychiatry* 1999; **45**: 7–12.

15 Beck AT, Steer RA, Brown GK. *Manual for the Beck Depression Inventory-II*. Psychological Corporation, 1996.

16 Kay SR, Fiszbein A, Opler LA. The Positive and Negative Syndrome Scale (PANSS) for Schizophrenia. *Schizophr Bull* 1987; **13**: 261–76.

17 Endicott J, Nee J, Harrison W, Blumenthal R. Quality of Life Enjoyment and Satisfaction Questionnaire: a new measure. *Psychopharmacol Bull* 1993; **29**: 321–6.

18 Addington D, Addington J, Schissel B. A depression rating scale for schizophrenics. *Schizophr Res* 1990; **3**: 247–51.

19 Hamilton M. A Rating Scale for Depression. *J Neurol Neurosurg Psychiatry* 1960; **23**: 56–62.

20 Hamilton M. The Assessment of Anxiety States by Rating. *Br J Med Psychol* 1959; **32**: 50–5.

21 Birchwood M, Smith J, Cochrane R, Wetton S, Copestake S. The Social Functioning Scale the Development and Validation of a New Scale of Social Adjustment for use in Family Intervention Programmes with Schizophrenic Patients. *Br J Psychiatry* 1990; **157**: 853–9.

22 Kirkpatrick B, Strauss GP, Nguyen L, Fischer BA, Daniel DG, Cienfuegos A, *et al.* The Brief Negative Symptom Scale: Psychometric Properties. *Schizophr Bull* 2011; **37**: 300–5.

23 Egger M, Davey Smith G, Schneider M, Minder C, Egger M, Davey Smith G, *et al.* Bias in meta-analysis detected by a simple, graphical test. *BMJ* 1997; **315**: 629–34.

24 Haddaway NR, Page MJ, Pritchard CC, McGuinness LA. PRISMA2020: An R package and Shiny app for producing PRISMA 2020-compliant flow diagrams, with interactivity for optimised digital transparency and Open Synthesis. *Campbell Syst Rev* 2022; **18**: e1230.
